# Synthesis and anticancer activity of multisubstituted purines and xanthines with one or two propynylthio and aminobutynylthio groups

**DOI:** 10.1007/s00044-018-2155-3

**Published:** 2018-03-06

**Authors:** Alicja Kowalska, Krystian Pluta, Małgorzata Latocha

**Affiliations:** 1 Department of Organic Chemistry, School of Pharmacy with the Division of Laboratory Medicine, The Medical University of Silesia, Jagiellońska 4, 41-200 Sosnowiec, Poland; 2 Department of Cell Biology, School of Pharmacy with the Division of Laboratory Medicine, The Medical University of Silesia, Jedności 8, 41-200 Sosnowiec, Poland

**Keywords:** Propynylthiopurines, Aminobutynylthiopurines, Aminobutynylthioxanthines, Anticancer activity, Structure–activity relationship

## Abstract

A synthesis of new 2,6-disubstituted and 2,6,8-trisubstituted 7-methylpurines as well as 8-substituted 3,7-dimethylxanthines containing a triple bond chain have been worked out. Purinethiones and xanthinethiones were converted into propynylthio derivatives, which were then further transformed via a Mannich reaction into aminobutynylthio derivatives (amine = pyrrolidine, piperidine, morpholine, and diethylamine). The products thus obtained represent various types of the purine and xanthine structure: 8-mono-, 2,6- and 6,8-dipropynylthio, 6- and 8-monoaminobutynylthio, 2,6- and 6,8-diaminobutynylthio derivatives. All of these compounds were tested for their anticancer activity against human glioblastoma SNB-19, human adenocarcinoma MDA-MB-231, and melanoma C-32 cell lines. The anticancer activity depends on the nature of the substituent and its localization in the purine and xanthine framework. Generally, compounds possessing two alkynylthio groups (propynylthio or aminobutynylthio) were more active than those possessing only one group. Some compounds exhibited stronger or similar anticancer activity to cisplatin. All compounds were also tested for their cytotoxic activity against normal human fibroblasts (HFF-1). The most promising anticancer compounds were found to be 2,6-dipropynylthio-7-methylpurine **4**, 2-chloro-6,8-dipropynylthio-7-methylpurine **14**, and 2-chloro-6,8-di(N-morpholinylbutynylthio)-7-methylpurine **15c** acting selectively on glioblastoma SNB-19, melanoma C-32, and adenocarcinoma MDA-MB-231 with the IC_50_ = 0.07–4.08 μg/mL.

## Introduction

Purine is a privileged heterocyclic nucleus existing in the chemical architecture of various bioactive compounds. It is an important pharmacophore interacting with the synthesis and function of nucleic acids and enzymes. Synthetically modified purines are inhibitors of protein kinase, fructose biphosphatase, adenylation enzyme, and adenosine receptor modulators (Bliman et al. 2014; Sharma et al. 2016).

The physicochemical properties of many substituted purine derivatives provide the potential for tight binding to deep hydrophobic folds of physiologically disease relevant proteins, such as thyrosine kinases, sulfatases, and phosphorylases. Purines could be the selectively target of these protein hydrophobic folds, influencing the progression of a disease (Riva-Toniolo et al. 2003).

Conventional thiopurines (6-mercaptopurine, 6-thioguanine, and azathioprine) are incorporated into the structure of natural biomolecules—they modify nucleic acid synthesis and are commonly used as effective anticancer and immunosuppressive drugs in the treatment of acute leukemia and autoimmune hepatitis (Prima et al. 2013; Deswal and Srivastava 2017). Thiopurines remain the first line of therapy in inflammatory bowel disease, Crohn’s disease, ulcerative colitis, and autoimmune hepatitis (Herreras and Iborra 2017; Ward et al. 2017; Warner et al. 2016).

6-Thiopurine derivatives containing the 1,2,3-triazole moiety present higher values regarding the inhibition of parasite multiplication than chloroquine and show antimalarial and antileishmanial activities (Corrales et al. 2011).

The pharmacologically active xanthines are commonly used for their effects as stimulants, bronchodilators, phosphodiesterase inhibitors, CFTR chloride-channel activators, and adenosine receptor antagonists. The selectivity of caffeine analogs for A_2A_ adenosine receptors was increased by the replacement of one or two methyl groups of caffeine with a propyl or propynyl substituent. 3,7-Dimethyl-1-propynylxanthine has been used as a selective A_2_ antagonist. 1-Propynyltheobromine and 1,3-dipropynyl-7-methylxanthine are approximately fourfold more potent than caffeine at the ryanodine sensitive calcium release channels, and fivefold more potent than caffeine as GABA_A_ receptor modulators. The range of clinical applications for xanthines has continued to widen and currently includes their use as anticonvulsants, nootropics, and therapeutics for the treatment of migraine (Daly et al. 2007; Thomas et al. 2013; Zagorska et al. 2015).

The introduction of substituents in positions 2, 6, and 9 of the purine ring enhances both binding affinity and selectivity regarding kinases. This enzyme family plays a critical role in the proliferation of human tumor cells, as well as in the survival and migration of neoplasia. There was an observed selectivity of 2-acetylenyl-6,9-disubstituted purine analogs regarding the inhibition of cyclin-dependent kinase (CDK1-cyclin B) (Legraverend et al. 2000; Sharma et al. 2016). The inhibitors of this enzyme play an important role in cell cycle regulation in a number of disease conditions, in particular cancer, and have been characterized as tumor suppressors. It is highly notable that the 2-nitrogen atom, which is characteristic of all other purine-based cyclin-dependent kinase inhibitors (CKIs), can be replaced by an *sp*-hybridized carbon atom, which effectively occupies the ribose binding pocket (Sharma et al. 2016).

Some substituents in positions 2 and 6 of the purine modulate both the affinity and selectivity of adenosine agonists for the different receptor subtypes. The 2-alkynyl adenosine and their N-ethylcarboxamide analogs, such as 2-hexynyl (HENECA) and 2-(R,S)-phenylhydroxypropynyl (R,S-PHPNECA), have proven to be highly potent A_2A_ adenosine receptor agonists (Volpini et al. 2002, 2005; Endo et al. 2014). The type of alkynyl chain in the 2 position seems to be very important for the potency at the A_2A_ receptor. Selective A_2A_ receptor subtype antagonists have emerged as an attractive target for Parkinson’s disease therapy as well as other neurodegenerative disorders such as schizophrenia and epilepsy. They are also effective as hypoglycemic agents (Baraldi et al. 1998; Endo et al. 2014). Apadenoson (ATL-146e) has entered phase III clinical trials as a pharmacologic stress agent for use in myocardial perfusion imaging (Foitzik et al. 2009).

Alkynyl chains in the 8 position of adenosine comprise a very selective ligand for the A_3_ receptor subtype. Thus, it is possible to modulate the activity at the adenosine receptor subtype by introduction of alkynyl chains in either the 2 or 8 positions of 9-ethyladenine (Volpini et al. 2001, 2005). 8-Substituted 2-alkynyl-N9-propynyladenine derivatives were found to antagonize the receptor and may be effective in the treatment of Parkinson’s disease (Endo et al. 2014; Ivachtchenko et al. 2013). 2-Propynyladenosinyl phenyl ether compositions as A_2A_ receptor agonists stimulate mammalian coronary vasodilatation and can be useful as therapeutics for coronary artery disease (Zablocki et al. 2001).

The presence of the alkynyl chain in 2-substituted adenosines is also very important for purinergic receptor pharmacology. Purine ionotropic receptors could serve as attractive targets for pharmacological interventions in various diseases. 2-Alkynyl derivatives of adenine, adenosine, N-alkylcarboxamidoadenosine, and adenine nucleotide are selective ligands for P2X ionotropic receptors (Koles et al. 2007; Dal Ben et al. 2011). 2-Hexynyl and 2-phenylethynyladenosine mono-, di-, and triphosphates are P2Y_1_ and P2Y_12_ receptor agonists and adenylyl cyclase modulators. Through interaction with P2Y_1_ and P2Y_12_ receptors, they effectively inhibit platelet aggregation and may be used in the prevention and treatment of arterial thrombosis as highly potent antithrombotic agents (Cristalli et al. 2005; Dal Ben et al. 2011).

Most 6-alkynyl-2-oxopurines and 6-phenylalkynylpurine nucleoside analogs exhibit cytotoxicity against a human chronic myelogenous leukemic cell line, K-562, that is comparable to or better than the known anticancer drugs, such as 6-mercaptopurine and fludarabine (Andersen et al. 2002; Brathe et al. 2003). The alkynyl-modified nucleosides substituted specifically in the 8 position, such as 8-alkynyladenosines and 2′-deoxyadenosines, proved to be very selective ligands for the A_3_ adenosine receptor subtype, and showed moderate anti-HCV and anti HIV-1 activity in cell culture (Kuchar et al. 2008; Dimopoulou et al. 2015). Oligonucleotides containing the alkynyl group in the 1 position of 6-thiopurine or 6-thioguanine moiety have demonstrated potent antiviral activity in several assays, including the human immunodeficiency virus (Broom et al. 1997). Some of the 2- or 6-alkynylpurine derivatives were prepared for use in antibacterial prevention and therapy (Atamanyuk et al. 2012). 6-Alkynylpurines also show profound inhibitory activity against human 15-lipoxygenase. The inhibitors of these enzymes have been implicated in the oxidation of low density lipoproteins, and could have potential as drugs against artherosclerosis and other major diseases, such as for instance cancer, Parkinson’s, Altzheimer’s disease, heart infraction, and rheumatoid arthritis, which are also linked to free radicals (Brathe et al. 2002; Berg et al. 2005). 6-Alkynylpurines as cytokine analogs also possess a profound plant growth stimulation effect (Brathe et al. 2003).

## Experimental

All commercially available organic solvents and reagents were from Sigma-Aldrich and Chempur and were used without further purification.

The melting points were determined in open capillary tubes on a Boetius melting point apparatus and were uncorrected. The ^1^H NMR and ^13^C NMR spectra were recorded on a Bruker Ascend^TM^ 600 spectrometer operating at 600 MHz and 150 MHz for ^1^H and ^13^C nuclei, respectively, in deuterochloroform and dimethyl sulphoxide-*d*_6_ with tetramethylsilane as internal standard. Shifts were given in ppm, coupling constant (*J*) values was presented in hertz (Hz), and the abbreviations were as follows: s (singlet), d (doublet), t (triplet), and m (multiplet). High resolution mass spectral analysis (HR ESIMS) was performed on a Bruker Impact II spectrometer (in the acetonitrile: 1% formic acid, 8:2 v/v). The reactions were monitored by thin layer chromatography (TLC) using sheets coated with silica gel 60_F254_ (Merck) and chloroform, ethanol (9:1 and 5:1) as the solvents. Purity of the synthesized compounds was confirmed by TLC on the same way. Spots were detected by their absorption under UV light (*λ* = 254 nm) and the chromatograms were further visualized by iodine vapor. Column chromatography separations were carried out with Merck Kieselgel 60 or aluminum oxide 90 (Merck) using a mixture of chloroform–ethanol (99:1, v/v) as an eluent.

2-Chloro-6-propynylthio-7-methylpurine **1**, (Kowalska et al. 2015), 7-methylpurine-2,6-dithione **3** (Kowalska 2007), and 2,6-dichloro-7-methylpurine **11** (Kowalska et al. 1985) were obtained according to the previously described method. Theobromine was commercially available from Sigma-Aldrich.

### Synthesis of 2-chloro-6-aminobut-2-ynyltio-7-methylpurines **2a**-c

2-Chloro-6-alkylaminobut-2-ynylthio-7-methylpurines **2a-c** were prepared from 6-propynylthio derivative **1** (0.12 g, 0.5 mmol) and amine (piperidine, morpholine, and diethylamine, 0.7 mmol) according to the previously described procedure (Kowalska et al. 2015).

### 2-Chloro-6-(4-N-piperidinylbut-2-ynylthio)-7-methylpurine **2a**

It was obtained as a pale yellow solid (0.14 g, 82%); mp 122–124 °C (EtOH); ^1^H NMR (CDCl_3_), *δ*: 1.44 (m, 2H, CH_2_), 1.65 (m, 4H, 2CH_2_), 2.54 (m, 4H, 2NCH_2_), 3.33 (s, 2H, SCH_2_), 4.13 (s, 3H, NCH_3_), 4.24 (t, *J* = 2.1 Hz, 2H, CCH_2_), 8.03 (s, 1H, H-8); (DMSO-d_6_), *δ*: 1.23 (m, 2H, CH_2_), 1.41 (m, 4H, 2CH_2_), 2.28 (m, 4H, 2NCH_2_), 3.20 (s, 2H, SCH_2_), 4.05 (s, 3H, NCH_3_), 4.25 (t, *J* = 2.4 Hz, 2H, CCH_2_), 8.60 (s, 1H, H-8), ^13^C NMR (DMSO-d_6_), *δ*: 18.86 (CH_2_, SCH_2_CC), 23.88 (CH_2_), 25.81 (2CH_2_), 34.87 (CH_3,_ NCH_3_), 47.44 (CH_2_, CCH_2_), 52.68 (2CH_2_, NCH_2_), 79.22 (C, SCH_2_CC), 80.34 (C, SCH_2_CC), 123.21 (C, C-5), 151.09 (CH, C-8), 152.38 (C, C-2), 154.27 (C, C-6), 160.55 (C, C-4); HR ESIMS m/z [M + H]^+^ calcd. for C_15_H_19_ClN_5_S 336.1050 found 336.1051.

### 2-Chloro-6-(4-N-morpholinylbut-2-ynylthio)-7-methylpurine **2b**

It was obtained as a pale yellow solid (0.16 g, 94%); mp 168–169 °C (EtOH); ^1^H NMR (CDCl_3_), *δ*: 2.62 (m, 4H, 2NCH_2_), 3.36 (s, 2H, SCH_2_), 3.78 (m, 4H, 2OCH_2_), 4.13 (s, 3H, NCH_3_), 4.25 (s, 2H, CCH_2_), 8.03 (s, 1H, H-8); (DMSO-d_6_), *δ*: 2.36 (m, 4H, 2NCH_2_), 3.24 (t, *J* = 2.4 Hz, 2H, SCH_2_), 3.52 (m, 4H, 2OCH_2_), 4.05 (s, 3H, NCH_3_), 4.27 (t, *J* = 2.4 Hz, 2H, CCH_2_), 8.59 (s, 1H, H-8); ^13^C NMR (DMSO-d_6_), *δ*: 18.74 (CH_2_, SCH_2_CC), 34.86 (CH_3,_ NCH_3_), 46.96 (CH_2_, CCH_2_), 51.95 (2CH_2_, NCH_2_), 66.45 (2CH_2_, OCH_2_), 78.82 (C, SCH_2_CC), 80.58 (C, SCH_2_CC), 123.12 (C, C-5), 151.09 (CH, C-8), 152.35 (C, C-2), 154.24 (C, C-6), 160.53 (C, C-4); HR ESIMS m/z [M + H]^+^ calcd. for C_14_H_17_ClN_5_OS 338.0842 found 338.0840.

### 2-Chloro-6-(4-diethylaminobut-2-ynylthio)-7-methylpurine **2c**

It was obtained as a pale yellow solid (0.14 g, 86%); mp 117–118 °C (EtOH); ^1^H NMR (CDCl_3_), *δ*: 1.06 (t, *J* = 7.2 Hz, 6H, 2CH_3_), 2.54 (q, *J* = 7.2 Hz, 4H, 2NCH_2_), 3.44 (s, 2H, SCH_2_), 4.13 (s, 3H, NCH_3_), 4.24 (t, *J* = 2.1 Hz, 2H, CCH_2_), 8.02 (s, 1H, H-8); (DMSO-d_6_), *δ*: 0.87 (t, *J* = 7.2 Hz, 6H, 2CH_3_), 2.32 (q, *J* = 7.2 Hz, 4H, 2NCH_2_), 3.33 (s, 2H, SCH_2_), 4.05 (s, 3H, NCH_3_), 4.24 (s, 2H, CCH_2_), 8.59 (s, 1H, H-8), ^13^C NMR (DMSO-d_6_), *δ*: 12.84 (2CH_3_, CH_2_CH_3_), 18.93 (CH_2_, SCH_2_CC), 34.86 (CH_3,_ NCH_3_), 40.74 (CH_2_, NCH_2_), 46.85 (2CH_2_, CCH_2_), 78.66 (C, SCH_2_CC), 80.10 (C, SCH_2_CC), 123.22 (C, C-5), 151.09 (CH, C-8), 152.38 (C, C-2), 154.26 (C, C-6), 160.55 (C, C-4); HR ESIMS m/z [M + H]^+^ calcd. for C_14_H_19_ClN_5_S 324.1050 found 324.1053.

### Synthesis of 2,6-di(prop-2-ynylthio)-7-methylpurine **4**

The mixture of 7-methylpurine-2,6-dithione **3** (0.10 g, 0.5 mmol) and potassium *tert*-butoxide (0.16 g, 1.44 mmol) in 10 mL of DMF was stirred at room temperature for 0.5 h. Then, 80% solution of propargyl bromide (0.17 g, 1.44 mmol) in dry toluene (0.25 mL) was added. The reaction mixture was stirred for an additional 24 h at room temperature and then poured into 25 mL of water. The resulted solid was filtered off and washed with water to give compound **4**.

It was obtained as a pale yellow solid (0.11 g, 79%); mp 162–164 °C (EtOH); ^1^H NMR (CDCl_3_), *δ*: 2.21 (t, *J* = 2.7 Hz, 1H, CH), 2.28 (t, *J* = 2.7 Hz, 1H, CH), 4.04 (d, *J* = 2.7 Hz, 2H, SCH_2_), 4.11 (s, 3H, NCH_3_), 4.22 (d, *J* = 2.7 Hz, 2H, SCH_2_), 8.09 (s, 1H, H-8); (DMSO-d_6_), *δ*: 3.14 (t, *J* = 2.4 Hz, 1H, CH), 3.26 (t, *J* = 2.4 Hz, 1H, CH), 4.02 (s, 3H, NCH_3_), 4.06 (d, *J* = 2.4 Hz, 2H, SCH_2_), 4.27 (d, *J* = 2.4 Hz, 2H, SCH_2_), 8.47 (s, 1H, H-8); ^13^C NMR (DMSO-d_6_), *δ*: 17.82 (CH_2_, SCH_2_CCH), 19.50 (CH_2_, SCH_2_CCH), 34.62 (CH_3_, NCH_3_), 73.56 (CH, SCH_2_CCH), 74.52 (CH, SCH_2_CCH), 80.09 (C, SCH_2_CCH), 81.24 (C, SCH_2_CCH), 121.42 (C, C-5), 149.92 (CH, C-8), 152.16 (C, C-2), 159.87 (C, C-6), 161.61 (C, C-4); HR ESIMS m/z [M + H]^+^ calcd. for C_12_H_11_N_4_S_2_ 275.0425 found 275.0417.

### General procedure for synthesis of 2,6-di(aminobut-2-ynylthio)-7-methylpurines **5a**-**d**

To a mixture of propynylthio derivative **2** (0.137 g, 0.5 mmol) and paraformaldehyde (0.067 g, 1.4 mmol) in 10 mL of dry dioxane, appropriate amines (pyrrolidine, piperidine, morpholine, or diethylamine, 1.4 mmol) and CuCl (0.01 g) were added. The reaction mixture was stirred at temperature 80 °C for 4 h. In the case of dimethylamine, the reaction was performed at 50 °C for 24 h. After cooling, the small amount of solid was filtered off and residue was extracted with chloroform (3 × 5 mL), dried with anhydrous Na_2_SO_4_, and evaporated in vacuo. The dried residue was dissolved in CHCl_3_ and purified by thin layer preparative chromatography (silica gel, CHCl_3_ – EtOH, 5:1 v/v) to give compound **5a**-**d**.

### **2,6**-**Di(4**-**N-pyrrolidinylbut-2-ynylthio)-7-methylpurine 5a**

It was obtained as a dark yellow solid (0.175 g, 79.5%); mp 97–99 °C (EtOH); ^1^H NMR (CDCl_3_), *δ*: 1.85 (m, 8H, 4CH_2_), 2.64 (m, 8H, 4NCH_2_), 3.44 (t, *J* = 2.1 Hz, 2H, SCH_2_), 3.47 (t, *J* = 2.1 Hz, 2H, SCH_2_), 3.98 (s, 3H, NCH_3_), 4.06 (m, 2H, CCH_2_), 4.21 (m, 2H, CCH_2_), 7.92 (s, 1H, H-8); (DMSO-d_6_), δ: 1.61 (m, 8H, 4CH_2_), 2.43 (m, 8H, 4NCH_2_), 3.36 (s, 4H, SCH_2_), 4.01 (s, 3H, NCH_3_), 4.10 (t, *J* = 1.8 Hz, 2H, CCH_2_), 4.30 (t, *J* = 1.8 Hz, 2H, CCH_2_), 8.46 (s, 1H, H-8); ^13^C NMR (DMSO-d_6_), *δ*: 18.41 (CH_2_, SCH_2_CC), 19.97(CH_2_, SCH_2_CC), 23.72 (4CH_2_), 34.64 (CH_3,_ NCH_3_), 42.70 (CH_2_, CCH_2_), 42.81 (CH_2_, CCH_2_), 52.01 (2CH_2_, NCH_2_), 52.07 (2CH_2_, NCH_2_), 78.36 (C, SCH_2_CC), 79.30 (C, SCH_2_CC), 80.23 (C, SCH_2_CC), 81.23 (C, SCH_2_CC), 121.42 (C, C-5), 149.85 (CH, C-8), 152.28 (C, C-2), 159.86 (C, C-6), 161.88 (C, C-4); HR ESIMS m/z [M + H]^+^ calcd. for C_22_H_29_N_6_S_2_ 441.1895 found 441.1886.

### 2,6-Di(4-N-piperidinylbut-2-ynylthio)-7-methylpurine **5b**

It was obtained as a dark yellow solid (0.18 g, 77%); mp 130–132 °C (EtOH); ^1^H NMR; (CDCl_3_), *δ*: 1.40 (m, 4H, 2CH_2_), 1.60 (m, 8H, 4CH_2_), 2.47 (m, 8H, 4NCH_2_), 3.28 (m, 4H, 2SCH_2_), 4.07 (s, 3H, NCH_3_), 4.07 (t, *J* = 2.1 Hz, 2H, CCH_2_), 4.23 (t, *J* = 2.1 Hz, 2H, CCH_2_), 7.92 (s, 1H, H-8); (DMSO-d_6_), *δ*: 1.23 (m, 4H, 2CH_2_), 1.43 (m, 8H, 4CH_2_), 2.30 (m, 8H, 4NCH_2_), 3.21 (m, 4H, 2SCH_2_), 4.01 (s, 3H, NCH_3_), 4.10 (s, 2H, CCH_2_), 4.32 (s, 2H, CCH_2_), 8.46 (s, 1H, H-8); ^13^C NMR (DMSO-d_6_), *δ*: 18.49 (CH_2_, SCH_2_CC), 20.05 (CH_2_, SCH_2_CC), 23.89 (CH_2_), 23.90 (CH_2_), 25.78 (2CH_2_), 25.80 (2CH_2_), 34.62 (CH_3,_ NCH_3_), 47.48 (CH_2_, CCH_2_), 47.53 (CH_2_, CCH_2_), 52.80 (4CH_2_, NCH_2_), 78.22 (C, SCH_2_CC), 79.15 (C, SCH_2_CC), 80.72 (C, SCH_2_CC), 81.72 (C, SCH_2_CC), 121.42 (C, C-5), 149.82 (CH, C-8), 152.31 (C, C-2), 159.86 (C, C-6), 161.90 (C, C-4); HR ESIMS m/z [M + H]^+^ calcd. for C_24_H_33_N_6_S_2_ 469.2208 found 469.2205.

### 2,6-Di(4-N-morpholinylbut-2-ynylthio)-7-methylpurine **5c**

It was obtained as a dark yellow solid (0.175 g, 74%); mp 121–123 °C (EtOH); ^1^H NMR (CDCl_3_), *δ*: 2.55 (m, 8H, 4NCH_2_), 3.30 (m, 4H, 2SCH_2_), 3.73 (m, 8H, 4OCH_2_), 4.08 (s, 3H, NCH_3_), 4.08 (t, *J* = 2.1 Hz, 2H, CCH_2_), 4.23 (t, *J* = 2.1 Hz, 2H, CCH_2_), 7.93 (s, 1H, H-8); (DMSO-d_6_), *δ*: 2.35 (m, 8H, 4NCH_2_), 3.23 (s, 2H, SCH_2_), 3.25 (s, 2H, SCH_2_), 3.52 (m, 8H, 4OCH_2_), 4.01 (s, 3H, NCH_3_), 4.11 (s, 2H, CCH_2_), 4.32 (s, 2H, CCH_2_), 8.46 (s, 1H, H-8); ^13^C NMR (DMSO-d_6_), *δ*: 18.39 (CH_2_, SCH_2_CC), 19.99 (CH_2_, SCH_2_CC), 34.63 (CH_3,_ NCH_3_), 47.03 (CH_2_, CCH_2_), 47.06 (CH_2_, CCH_2_), 52.00 (2CH_2_, NCH_2_), 52.09 (2CH_2_, NCH_2_), 66.47 (4CH_2_, OCH_2_), 77.77 (C, SCH_2_CC), 78.76 (C, SCH_2_CC), 80.95 (C, SCH_2_CC), 82.08 (C, SCH_2_CC), 121.43 (C, C-5), 149.83 (CH, C-8), 152.32 (C, C-2), 159.86 (C, C-6), 161.82 (C, C-4); HR ESIMS m/z [M + H]^+^ calcd. for C_22_H_29_N_6_O_2_S_2_ 473.1793 found 473.1795.

### 2,6-Di(4-diethylaminobut-2-ynylthio)-7-methylpurine **5d**

It was obtained as a dark yellow solid (0.173 g, 78%); mp 77–79 °C (EtOH); ^1^H NMR (CDCl_3_), *δ*: 1.06 (m, 12H, 4CH_3_), 2.54 (m, 8H, 4NCH_2_), 3.45 (m, 4H, 2SCH_2_), 4.07 (t, *J* = 2.1 Hz, 2H, CCH_2_), 4.08 (s, 3H, NCH_3_), 4.22 (t, *J* = 2.1 Hz, 2H, CCH_2_), 7.92 (s, 1H, H-8); (DMSO-d_6_), *δ*: 0.91 (m, 12H, 4CH_3_), 2.37 (m, 8H, 4NCH_2_), 3.35 (m, 4H, SCH_2_), 4.01 (s, 3H, NCH_3_), 4.09 (m, 2H, CCH_2_), 4.29 (m, 2H, CCH_2_), 8.46 (s, 1H, H-8); ^13^C NMR (DMSO-d_6_), *δ*: 12.80 (4CH_3_, CH_2_CH_3_), 17.80 (CH_2_, SCH_2_CC), 20.01 (CH_2_, SCH_2_CC), 34.62 (CH_3,_ NCH_3_), 40.84 (4CH_2_, NCH_2_), 46.92 (2CH_2_, CCH_2_), 74.52 (2C, SCH_2_CC), 80.03 (2C, SCH_2_CC), 121.42 (C, C-5), 149.91 (CH, C-8), 152.02 (C, C-2), 159.89 (C, C-6), 161.86 (C, C-4); HR ESIMS m/z [M + H]^+^ calcd. for C_22_H_33_N_6_S_2_ 445.2208 found 445.2204.

### Synthesis of 8-bromo-3,7-dimethylxanthine **7**

To a stirred suspension of theobromine **6 (**2.00 g, 10 mmol) in 4 mL of carbon tetrachloride and 12 mL of nitrobenzene, bromine (2.90 g, 0.9 mL, 18 mmol) dissolved in 1.5 mL of nitrobenzene was added dropwise. When the addition was completed, the reaction mixture was refluxed with stirring for 5 h. The suspension was then carefully poured with stirring into 40 mL of acetone. The resulting white precipitate was filtered, washed with acetone and ether, and dried in vacuo (Koppel et al. 1962). The crude product was purified by column chromatography (silica gel, CHCl_3_ - EtOH, 99:1, v/v) to give compound **7**.

It was obtained as a pale yellow solid (2.98 g, 69%), mp 298–300 °C, dec (EtOH), 298 °C dec (Koppel et al. 1962) ^1^H NMR (DMSO-d_6_), *δ*: 3.31 (s, 3H, N3CH_3_), 3.82 (s, 3H, N7CH_3_), 11.28 (s, 1H, N1H); ^13^C NMR (DMSO-d_6_), *δ*: 28.96 (CH_3_, N3CH_3_), 34.10 (CH_3_, N7CH_3_), 109.58 (C, C-5), 128.84 (CH, C-8), 149.48 (C, C-2), 151.04 (C, C-6), 154.58 (C, C-4); HR ESIMS m/z [M + H]^+^ calcd. for C_7_H_8_BrN_4_O_2_ 258.9831 found 258.9834.

### Synthesis of 3,7-dimethylxanthine-8-thione **8**

To the suspension of 8-bromo derivative **7** (0.26 g, 1 mmol) in 40 mL of anhydrous ethanol, sodium hydrosulfide (NaSH × 2 H_2_O, 0.92 g, 10 mmol) was added and the mixture was stirred and refluxed for 2 h. After cooling the alcohol was evaporated in vacuo and dry residue was dissolved in 5% NaOH solution. The reaction product was precipitated with 15% hydrochloric acid to give compound **8**.

It was obtained as a dark yellow solid (0.20 g, 95%), mp 322–324 °C (EtOH), 319–320 °C (Carson et al. 1998); ^1^H NMR (DMSO-d_6_), *δ*: 3.30 (s, 3H, N3CH_3_), 3.64 (s, 3H, N7CH_3_), 11.34 (s, 1H, N1H), 13.64 (s, 1H, N9H); ^13^C NMR (DMSO-d_6_), *δ*: 30.39 (CH_3_, N3CH_3_), 32.29 (CH_3_, N7CH_3_), 104.66 (C, C-5), 140.91 (CH, C-8), 150.12 (C, C-2), 153.11 (C, C-6), 164.19 (C, C-4); HR ESIMS m/z [M + H]^+^ calcd. for C_7_H_9_N_4_O_2_S 213.0446 found 213.0438.

### Synthesis of 8-(prop-2-ynylthio)-3,7-dimethylxanthine **9**

8-(Prop-2-ynylthio)-3,7-dimethylxanthine **9** was obtained from xanthine-8-thione **8** (0.11 g, 0.5 mmol) with propargyl bromide in DMF according to the previously described procedure (Kowalska et al. 2015).

It was obtained as a pale yellow solid (0.100 g, 80%); mp 252–253 °C (EtOH); ^1^H NMR (DMSO-d_6_), *δ*: 3.29 (t, *J* = 2.4 Hz, 1H, CH), 3.36 (s, 3H, N3CH_3_), 3.79 (s, 3H, N7CH_3_), 4.07 (d, *J* = 2.4 Hz, 2H, SCH_2_), 11.17 (s, 1H, NH); ^13^C NMR (DMSO-d_6_), *δ*: 21.64 (CH_2_, SCH_2_CCH), 28.99 (CH_3_, N3CH_3_), 32.79 (CH_3_, N7CH_3_), 75.26 (C, SCH_2_CCH), 79.93 (C, SCH_2_CCH), 109.21 (C, C-5), 148.19 (CH, C-8), 149.80 (C, C-2), 151.12 (C, C-6), 154.69 (C, C-4); HR ESIMS m/z [M + H]^+^ calcd. for C_10_H_11_N_4_O_2_S 251.0603 found 251.0600.

### Synthesis of 8-(aminobut-2-ynylthio)-3,7-dimethylxanthine **10a**-**d**

8-(Aminobut-2-ynylthio)-3,7-dimethylxanthines **10a-d** were obtained from 8-propynylthio derivative **9** (0.125 g, 0.5 mmol) and appropriate amines (pyrrolidine, piperidine, morpholine, and diethylamine, 0.7 mmol) in dry dioxane according to the described procedure (Kowalska et al. 2015).

### 8-(4-N-pyrrolidinylbut-2-ynylthio)-3,7-dimethylxanthine **10a**

It was obtained as a pale yellow solid (0.139 g, 84%); mp 195–197 °C (EtOH); ^1^H NMR (DMSO-d_6_), *δ*: 1.56 (m, 4H, 2CH_2_), 2.32 (m, 4H, 2NCH_2_), 3.33 (s, 2H, SCH_2_), 3.33 (s, 3H, N3CH_3_), 3.80 (s, 3H, N7CH_3_), 4.07 (t, *J* = 2.4 Hz, 2H, CCH_2_), 11.19 (s, 1H, NH); ^13^C NMR (DMSO-d_6_), *δ*: 22.36 (CH_2_, SCH_2_CC), 23.65 (2CH_2_), 28.95 (CH_3,_ N3CH_3_), 32.83 (CH_3,_ N7CH_3_), 42.48 (CH_2_, CCH_2_), 51.73 (2CH_2_, NCH_2_), 80.10 (C, SCH_2_CC), 80.15 (C, SCH_2_CC), 109.11 (C, C-5), 148.28 (CH, C-8), 149.82 (C, C-2), 151.11 (C, C-6), 154.68 (C, C-4); HR ESIMS m/z [M + H]^+^ calcd. for C_15_H_20_N_5_O_2_S 334.1338 found 334.1343.

### 8-(4-N-piperidinylbut-2-ynylthio)-3,7-dimethylxanthine **10b**

It was obtained as a pale yellow solid (0.120 g, 69%); mp 223–225 °C (EtOH); ^1^H NMR (DMSO-d_6_), *δ*: 1.23 (m, 2H, CH_2_), 1.39 (m, 4H, 2CH_2_), 2.19 (m, 4H, 2NCH_2_), 3.18 (t, *J* = 1.8 Hz, 2H, SCH_2_)_,_ 3.32 (s, 3H, N3CH_3_), 3.81 (s, 3H, N7CH_3_), 4.08 (t, *J* = 1.8 Hz, 2H, CCH_2_), 11.19 (s, 1H, NH); ^13^C NMR (DMSO-d_6_), *δ*: 22.42 (CH_2_, SCH_2_CC), 23.90 (CH_2_), 25.81 (2CH_2_), 28.95 (CH_3,_ N3CH_3_), 32.87 (CH_3,_ N7CH_3_), 47.37 (CH_2_, CCH_2_), 52.57 (2CH_2_, NCH_2_), 79.92 (C, SCH_2_CC), 80.62 (C, SCH_2_CC), 109.12 (C, C-5), 148.25 (CH, C-8), 149.84 (C, C-2), 151.11 (C, C-6), 154.69 (C, C-4); HR ESIMS m/z [M + H]^+^ calcd. for C_16_H_22_N_5_O_2_S 348.1494 found 348.1489.

### 8-(4-N-morpholinylbut-2-ynylthio)-3,7-dimethylxanthine **10c**

It was obtained as a pale yellow solid (0.126 g, 72%); mp 174–176 °C (EtOH); ^1^H NMR (DMSO-d_6_), *δ*: 2.24 (m, 4H, 2NCH_2_), 3.24 (t, *J* = 1.8 Hz, 2H, SCH_2_), 3.33 (s, 3H, N3CH_3_), 3.81 (s, 3H, N7CH_3_), 4.09 (t, *J* = 1.8 Hz, 2H, CCH_2_), 4.47 (t, *J* = 1.8 Hz, 4H, 2OCH_2_), 11.15 (s, 1H, NH); ^13^C NMR (DMSO-d_6_), *δ*: 22.30 (CH_2_, SCH_2_CC), 28.96 (CH_3,_ N3CH_3_), 32.86 (CH_3,_ N7CH_3_), 46.80 (CH_2_, CCH_2_), 51.53 (2CH_2_, NCH_2_), 66.43 (2CH_2_, 2OCH_2_), 79.28 (C, SCH_2_CC), 81.15 (C, SCH_2_CC), 109.18 (C, C-5), 148.13 (CH, C-8), 149.86 (C, C-2), 151.15 (C, C-6), 154.73 (C, C-4); HR ESIMS m/z [M + H]^+^ calcd. for C_15_H_20_N_5_O_3_S 350.1287 found 350.1279.

### 8-(4-diethylaminobut-2-ynylthio)-3,7-dimethylxanthine **10d**

It was obtained as a pale yellow solid (0.131 g, 78%); mp 186–188 °C (EtOH); ^1^H NMR (DMSO-d_6_), *δ*: 0.86 (t, *J* = 7.2 Hz, 6H, 2CH_3_), 2.24 (q, *J* = 7.2 Hz, 4H, 2NCH_2_), 3.30 (s, 2H, SCH_2_)_,_ 3.30 (s, 3H, N3CH_3_), 3.80 (s, 3H, N7CH_3_), 4.07 (s, 2H, CCH_2_), 11.18 (s, 1H, NH); ^13^C NMR (DMSO-d_6_), *δ*: 12.82 (2CH_3_,), 22.43 (SCH_2_CC), 28.94, (CH_3,_ N3CH_3_), 32.85 (CH_3,_ N7CH_3_), 40.69 (CH_2_, CCH_2_), 46.71 (2CH_2_, NCH_2_), 79.33 (C, SCH_2_CC), 80.36 (C, SCH_2_CC), 109.10 (C, C-5), 148.26 (CH, C-8), 149.85 (C, C-2), 151.12 (C, C-6), 154.67 (C, C-4); HR ESIMS m/z [M + H]^+^ calcd. for C_15_H_22_N_5_O_2_S 336.1494 found 336.1491.

### Synthesis of 2-chloro-6,8-dibromo-7-methylpurine **12**

To a stirred suspension of 2,6-dichloro-7-methylpurine **11** (2.00 g, 9.8 mmol) in 4 mL of carbon tetrachloride and 12 mL of nitrobenzene, bromine (4.90 g, 1.6 mL, 31 mmol) dissolved in 3.0 mL of nitrobenzene was added dropwise. When the addition was completed, the reaction mixture was refluxed with stirring for 5 h. The suspension was then carefully poured with stirring into 40 mL of acetone. The resulting white precipitate was filtered, washed with acetone and ether, and dried in vacuo. Crude product was purified by column chromatography (silica gel, CHCl_3_ - ETOH, 99:1, v/v) to give compound **12**.

It was obtained as a pale yellow solid (2.15 g, 67%), mp 218–220 °C, (EtOH); ^1^H NMR (DMSO-d_6_), *δ*: 4.02 (s, 3H, N7CH_3_), ^13^C NMR (DMSO-d_6_), *δ*: 35.22 (CH_3_, NCH_3_), 127.07 (C, C-5), 133.88 (CH, C-8), 142.90 (C, C-2), 151.46 (C, C-6), 161.03 (C, C-4); HR ESIMS m/z [M + H]^+^ calcd. for C_6_H_4_Br_2_ClN_4_ 324.8491 found 324.8489.

### Synthesis of 2-chloro-7-methylpurine-6,8-dithione **13**

A solution of 6,8-dibromo derivative **12** (0.326 g, 1 mmol) and sodium hydrosulfide (NaSH × 2H_2_O, 0.92 g, 10 mmol) in 40 mL ethanol was refluxed for 2 h. The alcohol was evaporated in vacuo and dry residue was dissolved in 5% NaOH solution. The reaction product was precipitated with 15% hydrochloric acid to give compound **13**.

It was obtained as a dark yellow solid (0.225 g, 97%), mp 296–298 °C (EtOH); ^1^H NMR (DMSO-d_6_), *δ*: 4.03 (s, 3H, N7CH_3_), 13.88 (s, 1H, N1H), 13.91 (s, 1H, N9H); ^13^C NMR (DMSO-d_6_), *δ*: 34.62 (CH_3_, NCH_3_), 121.42 (C, C-5), 149.92 (CH, C-8), 152.16 (C, C-2), 159.87 (C, C-6), 161.61 (C, C-4); HR ESIMS m/z [M + H]^+^ calcd. for C_6_H_6_ClN_4_S_2_ 232.9722 found 232.9720.

### Synthesis of 2-chloro-6,8-di(prop-2-ynylthio)-7-methylpurine **14**

2-Chloro-6,8-di(prop-2-ynylthio)**-**7-methylpurine **14** was obtained by alkylation of purine-6,8-dithione **13** (0.12 g, 0.5 mmol) with propargyl bromine in DMF according to the procedure described for the synthesis compound **4**.

It was obtained as a pale yellow solid (0.105 g, 68%); mp 161–163 °C (EtOH), ^1^H NMR (DMSO-d_6_), *δ*: 3.26 (t, *J* = 2.4 Hz, 1H, CH), 3.31 (t, *J* = 2.4 Hz, 1H, CH), 3.88 (s, 3H, NCH_3_), 4.22 (d, *J* = 2.4 Hz, 2H, SCH_2_), 4.26 (d, *J* = 2.4 Hz, 2H, SCH_2_); ^13^C NMR (DMSO-d_6_), *δ*: 18.22 (CH_2_, SCH_2_CCH), 21.02 (CH_2_, SCH_2_CCH), 33.90 (CH_3_, NCH_3_), 74.68 (CH, SCH_2_CCH), 75.05 (CH, SCH_2_CCH), 79.61 (C, SCH_2_CCH), 79.68 (C, SCH_2_CCH), 125.50 (C, C-5), 150.98 (CH, C-8), 152.08 (C, C-2), 159.62 (C, C-6), 159.97 (C, C-4); HR ESIMS m/z [M + H]^+^ calcd. for C_12_H_10_ClN_4_S_2_ 309.0035 found 309.0032.

### Synthesis of 2-chloro-6,8-di(aminobut-2-ynylthio)-7-methylpurines **15a**-**d**

2-Chloro-6,8-di(aminobut-2-ynylthio)-7-methylpurines **15a**-**d** were obtained from 6,8-dipropynylthio derivative **14** (0.15 g, 0.5 mmol) and appropriate amines ((pyrrolidine, piperidine, morpholine, and diethylamine, 0.7 mmol) in dry dioxane according to the previously described procedure for the synthesis of 2,6-di(aminobut-2-ynylthio)-7-methylpurines **5a**-**d**.

### 2-Chloro-6,8-di(4-N-pyrrolidinylbut-2-ynylthio)-7-methylpurine **15a**

It was obtained as a pale yellow solid (0.183 g, 77%); mp 133–135 °C (EtOH); ^1^H NMR (DMSO-d_6_), *δ*: 1.58 (m, 8H, 4CH_2_), 2.38 (m, 8H, 4NCH_2_), 3.34 (t, *J* = 2.4 Hz, 2H, SCH_2_), 3.36 (t, *J* = 2.4 Hz, 2H, SCH_2_), 3.88 (s, 3H, NCH_3_), 4.22 (t, *J* = 2.4 Hz, 2H, CCH_2_), 4.27 (t, *J* = 2.4 Hz, 2H, CCH_2_); ^13^C NMR (DMSO-d_6_), *δ*: 18.93 (CH_2_, SCH_2_CC), 18.96 (CH_2_, SCH_2_CC), 21.70 (CH_3,_ NCH_3_), 23.66 (2CH_2_), 23.71 (2CH_2_), 33.91 (CH_2_, CCH_2_), 33.92 (CH_2_, CCH_2_), 42.57 (CH_2_, NCH_2_), 42.63 (CH_2_, NCH_2_), 51.83 (CH_2_, NCH_2_), 51.95 (CH_2_, NCH_2_), 79.32 (C, SCH_2_CC), 79.38 (C, SCH_2_CC), 79.89 (C, SCH_2_CC), 80.03 (C, SCH_2_CC), 125.52 (C, C-5), 143.07 (CH, C-8), 151.20 (C, C-2), 152.19 (C, C-6), 159.84 (C, C-4); HR ESIMS m/z [M + H]^+^ calcd. for C_22_H_28_ClN_6_S_2_ 475.1505 found 475.1491.

### 2-Chloro-6,8-di(4-N-piperidinylbut-2-ynylthio)-7-methylpurine **15b**

It was obtained as a pale yellow solid (0.165 g, 74%); mp 108–110 °C (EtOH); ^1^H NMR (DMSO-d_6_), *δ*: 1.23 (m, 4H, 2CH_2_), 1.40 (m, 8H, 4CH_2_), 2.26 (m, 8H, 4NCH_2_), 3.19 (s, 4H, 2SCH_2_), 3.89 (s, 3H, NCH_3_), 4.23 (t, *J* = 2.4 Hz, 2H, CCH_2_), 4.28 (t, *J* = 2.4 Hz, 2H, CCH_2_); ^13^C NMR (DMSO-d_6_), *δ*: 18.97 (2CH_2_, SCH_2_CC), 21.77 (CH_3,_ NCH_3_), 23.85 (CH_2_), 23.88 (CH_2_), 25.76 (2CH_2_), 25.81 (2CH_2_), 33.94 (CH_2_, CCH_2_), 33.95 (CH_2_, CCH_2_), 47.41 (CH_2_, NCH_2_), 47.43 (CH_2_, NCH_2_), 52.67 (CH_2_, NCH_2_), 52.71 (CH_2_, NCH_2_), 79.14 (C, SCH_2_CC), 79.75 (C, SCH_2_CC), 80.38 (C, SCH_2_CC), 80.43 (C, SCH_2_CC), 125.54 (C, C-5), 143.10 (CH, C-8), 151.24 (C, C-2), 152.22 (C, C-6), 159.81 (C, C-4); HR ESIMS m/z [M + H]^+^ calcd. for C_24_H_32_ClN_6_S_2_ 503.1818 found 503.1814.

### 2-Chloro-6,8-di(4-N-morpholinylbut-2-ynylthio)-7-methylpurine **15c**

It was obtained as a pale yellow solid (0.172 g, 70%); mp 112–113 °C (EtOH); ^1^H NMR (DMSO-d_6_), *δ*: 2.30 (m, 4H, 2NCH_2_), 2.35 (m, 4H, 2NCH_2_), 3.23 (m, 4H, SCH_2_), 3.47 (m, 4H, 2OCH_2_), 3.51 (m, 4H, 2OCH_2_), 3.89 (s, 3H, NCH_3_), 4.25 (t, *J* = 2.4 Hz, 2H, CCH_2_), 4.29 (t, *J* = 2.4 Hz, 2H, CCH_2_); ^13^C NMR (DMSO-d_6_), *δ*: 18.89 (CH_2_, SCH_2_CC), 18.91 (CH_2_, SCH_2_CC), 21.73 (CH_3,_ NCH_3_), 33.93 (CH_2_, CCH_2_), 33.94 (CH_2_, CCH_2_), 46.90 (CH_2_, NCH_2_), 46.96 (CH_2_, NCH_2_), 51.85 (CH_2_, 2NCH_2_), 66.46 (CH_2_, 4OCH_2_), 78.83 (C, SCH_2_CC), 79.33 (C, SCH_2_CC), 80.62 (C, SCH_2_CC), 80.74 (C, SCH_2_CC), 125.51 (C, C-5), 143.05 (CH, C-8), 151.23 (C, C-2), 152.18 (C, C-6), 159.95 (C, C-4); HR ESIMS m/z [M + H]^+^ calcd. for C_22_H_28_ClN_6_O_2_S_2_ 507.1404 found 507.1398.

### 2-Chloro-6,8-di(diethylaminobut-2-ynylthio)-7-methylpurine **15d**

It was obtained as a pale yellow solid (0.194 g, 81%); mp 88–90 °C (EtOH); ^1^H NMR (DMSO-d_6_), *δ*: 0.87 (m, 12H, 4CH_3_), 2.32 (m, 8H, 4NCH_2_), 3.33 (s, 4H, 2SCH_2_), 3.88 (s, 3H, NCH_3_), 4.22 (t, *J* = 2.4 Hz, 2H, CCH_2_), 4.27 (t, *J* = 2.4 Hz, 2H, CCH_2_); ^13^C NMR (DMSO-d_6_), *δ*: 12.76 (4CH_3_,), 19.06 (CH_2_, SCH_2_CC), 19.09 (CH_2_, SCH_2_CC), 21.82 (CH_3,_ NCH_3_), 33.93 (CH_2_, CCH_2_), 33.94 (CH_2_, CCH_2_), 46.83 (CH_2_, 2NCH_2_), 46.85 (CH_2_, 2NCH_2_), 78.41 (C, SCH_2_CC), 79.17 (C, SCH_2_CC), 80.19 (C, SCH_2_CC), 80.36 (C, SCH_2_CC), 125.97 (C, C-5), 143.09 (CH, C-8), 151.22 (C, C-2), 152.22 (C, C-6), 159.81 (C, C-4); HR ESIMS m/z [M + H]^+^ calcd. for C_22_H_32_ClN_6_S_2_ 479.1818 found 479.1813.

### Antiproliferative assay in vitro

#### Cell culture

Compounds were evaluated for their anticancer activity using three cultured cell lines: SNB-19 (human glioblastoma, DSMZ - German Collection of Microorganisms and Cell Cultures, Braunschweig, Germany), C-32 (human amelanotic melanoma, ATCC-American Type Culture Collection, Manassas, VA, USA), MDA-MB-231 (human adenocarcinoma mammary gland, ATCC, Manassas, VA, USA), and HFF-1 (human fibroblast cell line, ATCC, Manassas, VA, USA). The cultured cells were kept at 37 °C and 5% CO_2_. The cells were seeded (1 × 10^4^ cells/well/100 μL DMEM supplemented with 10% FCS and streptomycin and penicillin) using 96-well plates (Corning). The cells were counted in a hemocytometer (Burker’s chamber) using a phase contrast Olympus IX50 microscope equipped with Sony SSC-DC58 AP camera and Olympus DP10 digital camera.

#### Proliferation assay

The antiproliferative effect of the compounds obtained from both the cancer and the normal cells was determined using the Cell Proliferation Reagent WST-1 assay (Roche Diagnostics, Mannheim, Germany). This colorimetric assay is based on the viable cell’s ability to cause the bright red-colored stable tetrazolium salt (2-(4-iodophenyl)-3-(4-nitrophenyl)-5-(2,4-disulfophenyl)-2H-tetrazolium, monosodium salt) to cleave to the dark red soluble formazan by cellular enzymes. An expansion in the number of viable cells results in an increase in the overall activity of mitochondrial dehydrogenases in the sample. An increase in the amount of formazan dye formed correlates to the number of metabolically active cells in the culture. The formazan dye produced by metabolically active cells is quantified by a scanning ELISA reader that measures the absorbance of the dye solution at appropriate wavelengths. The examined cells were exposed to the tested compounds for 72 h at various concentrations between 0.1 and 100 μg/mL (prepared initially at a concentration of 1 mg/mL in DMSO). The control was performed in order to check that DMSO has no effect on the cells at the concentration used. The cells were incubated with WST-1 (10 μL) for 1 h and the absorbance of the samples was measured against a background control at 450 nm using a microplate reader with a reference wavelength at 600 nm. The results are expressed as the means of at least two independent experiments performed in triplicate. The antiproliferative activity of the tested compound was compared to cisplatin. The IC_50_ values (a concentration of a compound that is required for 50% inhibition) were calculated from the dose–response relationship with respect to control.

## Result and discussion

### Chemistry

2-Chloro-6-(prop-2-ynylthio)-7-methylpurine **1** was obtained via S-alkylation of the appropriate 6-purinethione with propargyl bromide (as solution in toluene) in DMF at room temperature in the presence of potassium *tert*-butoxide (Kowalska et al. 2015). Using this procedure, 2,6-dipropynylthio-7-methylpurine **4** was obtained from 2,6-purinethione **3**. 2-Chloro-6-propynylthio-7-methylpurine **1** and 2,6-dipropynylthio-7-methylpurine **4**, which were transformed via a Mannich reaction with paraformaldehyde and secondary amine (pyrrolidine, piperidine, morpholine, and diethylamine) in dry dioxane in the presence of catalytic amounts of CuCl into 2-chloro-6-aminobut-2-ynylthio-7-methylpurines **2a**-**c**, as well as 2,6-di(aminobut-2-ynylthio)-7-methylpurines **5a**-**d** in yields of 82–94% and 74–79.5%, respectively. Whereas 2- and 6-chloropurines are easily obtained from oxopurines, the introduction of the halogen atom in the 8 position requires halogenation with bromine (Koppel et al. 1962). Compounds **6** and **11** were brominated with bromine (as a solution in nitrobenzene) followed by a refluxing of the reaction mixture for 5 h. In the case of compound **11**, the bromination proceeded not only in the 8 position but also in ipso in the 6 position. 8-Xanthinethione **8** and 8-purinethione **13** were obtained in the sodium hydrosulfide reaction. In order to obtain 8-aminobutynylthioxanthines **10a**-**d** and 6,8-diaminobutynylthiopurines **15a**-**d**, the same sequence of propynylation and a Mannich reaction was used.

Scheme 1

**Scheme 1:**
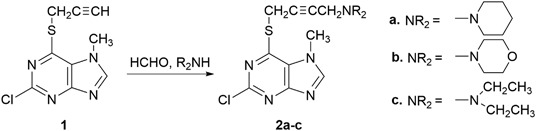
Synthesis of 2-chloro-6-aminobutynylthio-7-methylpurines **2a-c**

Scheme 2

**Scheme 2:**
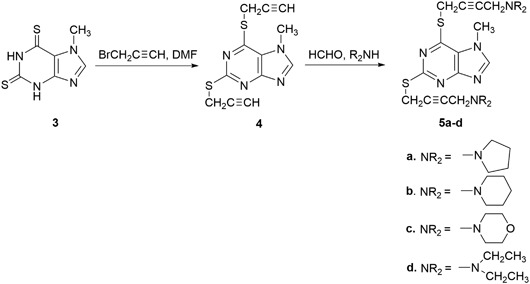
Synthesis of 2,6-dipropynylthio- and 2,6-diaminobutynylthio-7-methylpurines **4** and **5a-d**

Scheme 3

**Scheme 3:**
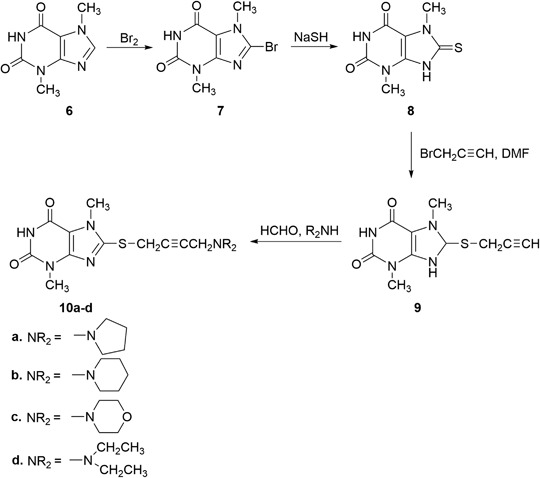
Synthesis of 8-propynylthio- and 8-aminobutynylthio-3,7-dimethylxanthines **9** and **10a-d**

Scheme 4

**Scheme 4:**
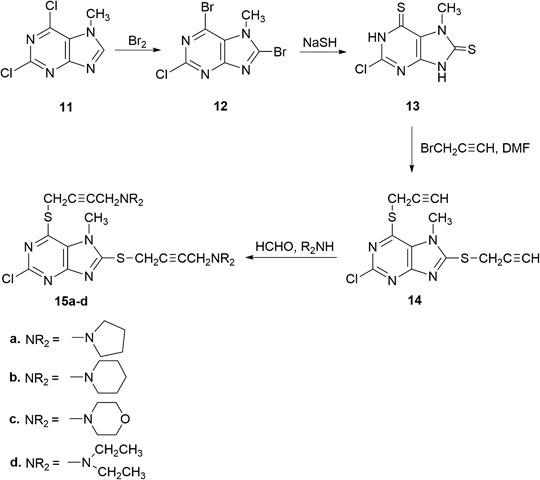
Synthesis of 2-chloro-6,8-dipropynylthio- and 2-chloro-6,8-diaminobutynylthio-7-methylpurines **14** and **15a**-**d**

### Anticancer activity

For the biological tests, 18 propynylthio- and aminobutynylthiopurines and xanthines of 4 types were selected:

a. 2-chloro-6-aminobutynylthio derivatives containing the piperidinyl, morpholinyl, and diethylamino groups (**2a**-**c**),

b. 2,6-dipropynylthio (**4**) and 2,6-diaminobutynylthiopurines containing the pyrrolidinyl, piperidinyl, morpholinyl, and diethylamino groups (**5a**-**d**),

c. 8-propynylthio (**9**) and 8-aminobutynylthioxanthines derivatives containing the pyrrolidinyl, piperidinyl, morpholinyl, and diethylamino groups (**10a**-**d**),

d. the 2-chloro-6,8-dipropynylthio (**14**) and 2-chloro-6,8-diaminobutynylthiopurines containing pyrrolidinyl, piperidinyl, morpholinyl, and diethylamino groups (**15a**-**d**),

The anticancer activity of the compounds obtained was investigated in vitro using cultured human glioblastoma SNB-19, human adenocarcinoma MDA-MB-231, and melanoma C-32 cell lines. Normal human fibroblasts (HFF-1) were used as a control and cisplatin was used as a reference drug. Table 1 shows the activity of alkynylthiopurines and xanthines **2a**-**c**, **4**, **5a**-**d**, **9**, **10a**-**d**, **14**, and **15a**-**d** as the IC_50_ values. The tested compounds exhibited different activities against three cancer cell lines. The SNB-19 and C-32 lines were more sensitive than the MDA-MB-231 line for most compounds.

**Table 1:** Antiproliferative activity of alkynylthiopurines and xanthines

Compounds	Antiproliferative activity IC_50_ (μg/mL)			
	SNB-19	C-32	MDA-MB-231	HFF-1
**2a**	28.80	21.01	33.40	35.42
**2b**	35.40	125.00	66.28	20.09
**2c**	17.40	11.88	48.06	8.65
**4**	15.01	1.77	1.00	10.00
**5a**	19.41	6.81	19.95	10.00
**5b**	4.64	6.39	7.35	3.98
**5c**	21.54	0.35	50.80	0.37
**5d**	48.93	23.86	38.49	5.95
**9**	191.10	103.46	169.34	153.65
**10a**	176.62	51.08	471.50	34.26
**10b**	166.17	355.00	363.50	30.39
**10c**	1.69	222.72	6.54	5.87
**10d**	1.48	26.36	68.12	3.68
**14**	0,07	270.35	10.00	20.09
**15a**	0.52	4.55	3.83	3.63
**15b**	11.62	19.08	38.07	5.50
**15c**	26.10	4.08	74.05	36.38
**15d**	2.91	5.33	10.00	1.00
Cisplatin	1.46	5.71	11.85	1.21

As shown in Table 1, 6,8-disubstituted derivatives **14** and **15a-d** were the most active group of the tested compounds and in some cases were more or at least as active as cisplatin. 2-Chloro-6,8-dipropynylthiopurine **14** and its pyrrolidinylmethyl derivative **15a** exhibited strong antiproliferative activity (IC_50_ values of <1 μg/mL) against the SBN-19 line. The last compound was also active against two other lines (IC_50_ = 3.8–4.5 μg/mL). Compounds **15c** and **15d** (containing two morpholinylbutynylthio and diethylaminobutynylthio groups) showed selective activity against the SNB-19 and C-32 lines (IC_50_ = 2.9–5.3 μg/mL).

The activity of 2,6-disubstituted compounds (**4** and its aminomethyl derivatives **5a**-**c**) were also selective. 2,6-Dipropynylthiopurine **4** was more active than cisplatin against the MDA-MB-231 and C-32 cell lines (IC_50_ ≤ 1.77 μg/mL). The introduction of the pyrrolidinylmethyl and piperidinylmethyl moieties to the propynylthio chain (compounds **5a** and **5b**) slightly decreased activity against selected lines. On the other hand, derivative **5c** (the morpholinylmethyl moiety that was introduced) was very active (more so than cisplatin) with the IC_50_ value of 0.35 μg/mL against the C-32 cell line.

2-Chloro-6-aminobutynyltiopurines **2a-c** were less active against all cell lines with IC_50_ values over 11.88 μg/mL.

The other monoalkynylthiosubstituted derivatives **9** and **10a**-**d** containing the 3,7-dimethylxanthine framework showed very different activity. In contrast to the very active 2,6- and 6,8-dipropynylthio derivatives **4** and **14**, 8-propynylthio derivative **9** was found inactive (IC_50_ > 100 μg/mL). Surprisingly, its derivatives **10d** and **10c** (containing the diethylaminobutynylthio and morpholinylbutynylthio groups) exhibited strong activity against the SBN-19 line (IC_50_ < 1.7 μg/mL). Two other derivatives (**10a** and **10b**) were found rather inactive.

All the compounds were tested for their cytotoxicity on the normal human fibroblasts (HFF-1). Some compounds (**5b**-**d**, **10c**-**d**, **15a**-**b**, and **15d**) as well as cisplatin also turned out to be cytotoxic for fibroblasts. Very active compounds **4** (against adenocarcinoma MDA-MB-231 and melanoma C-32), **14** (against glioblastoma SNB-19), and **15c** (against melanoma C-32) were found to have relatively weak cytotoxicity.

## Conclusion

While searching for alkynylthiopurines and aminoalkynylthiopurines and xanthines with the anticancer activity, we worked out a synthesis of 18 new multisubstituted 7-methylpurines and 3,7-dimethylxanthines containing one or two propynylthio groups (in 2, 6, and 8 positions), which were further transformed via a Mannich reaction with cyclic and open chain amines and formaldehyde into aminobutynylthio derivatives. The products obtained represent various types of the purine and xanthine structure containing the triple bond: 8-mono-, 2,6-, and 6,8-dipropynylthio derivatives **4**, **9**, and **14**, 6- and 8-monoaminobutynylthio derivatives **2a**-**c** and **10a**-**d**, 2,6- and 6,8-diaminobutynylthio derivatives **5a**-**d** and **15a**-**d**. All tested compounds exhibited different anticancer activity against human glioblastoma SNB-19, human adenocarcinoma MDA-MB-231, and melanoma C-32 cell lines depending on the nature of the substituent and its localization in the purine framework. Some compounds exhibited stronger or similar anticancer activity to cisplatin. A small number of compounds were also cytotoxic against the normal human fibroblasts (HFF-1). The most promising compounds with strong anticancer activity and relatively low cytotoxicity turned out to be 2,6-dipropynylthio-7-methylpurine **4**, 2-chloro-6,8-dipropynylthio-7-methylpurine **14**, and 2-chloro-6,8-di(N-morpholinylbutynylthio)-7-methylpurine **15c**.

## Electronic supplementary material

Supplementary Material
