# Supplementary material for: Synthesis and anticancer activity of multisubstituted purines and xanthines with one or two propynylthio and aminobutynylthio groups
Source: Med Chem Res. 2018 Mar 6;27(5):1384–95. doi: 10.1007/s00044-018-2155-3 (PMC5904222; doi:10.1007/s00044-018-2155-3)

Supplementary Material

SYNTHESIS AND ANTICANCER ACTIVITY OF MULTISUBSTITUTED PURINES AND XANTHINES WITH ONE OR TWO PROPYNYLTHIO AND AMINOBUTYNYLTHIO GROUPS.

Alicja Kowalska[1]*, Krystian Pluta[1] and Małgorzata Latocha[2]

[1]The Medical University of Silesia, School of Pharmacy with the Division of Laboratory Medicine, Department of Organic Chemistry, Jagiellońska 4, 41-200 Sosnowiec, Poland,

[2]The Medical University of Silesia, School of Pharmacy with the Division of Laboratory Medicine, Department of Cell Biology, Jedności 8, 41-200 Sosnowiec, Poland

E-mail: kowalska@sum.edu.pl

Content

The HR ESIMS, ^{1}H and ^{13}C NMR spectra

1. Compound **2a**
2 Compound **2b**
3. Compound **2c**
4. Compound **4**
5. Compound **5a**
6. Compound **5b**
7. Compound **5c**
8. Compound **5d**
9. Compound **9**
10. Compound **10a**
11. Compound **10b**
12. Compound **10c**
13. Compound **10d**
14. Compound **14**
15. Compound **15a**
16. Compound **15b**
17. Compound **15c**
18. Compound **15d**

Compound **2a** HR ESIMS

Mass Spectrum List Report

Analysis Info

Analysis Name	D:\Data\AK77-1.d	Acquisition Date	6/1/2017 1:12:00 PM
Method	low_mass.m	Operator	KM
Sample Name	TM Low concentration	Instrument	impact II 1825265.10082
Comment			

Acquisition Parameter

Source Type	ESI	Ion Polarity	Positive	Set Nebulizer	0.3 Bar
Focus	Active	Set Capillary	4000 V	Set Dry Heater	240 °C
Scan Begin	100 m/z	Set End Plate Offset	-500 V	Set Dry Gas	4.0 l/min
Scan End	1000 m/z	Set Charging Voltage	2000 V	Set Divert Valve	Source
		Set Corona	0 nA	Set APCI Heater	0 °C

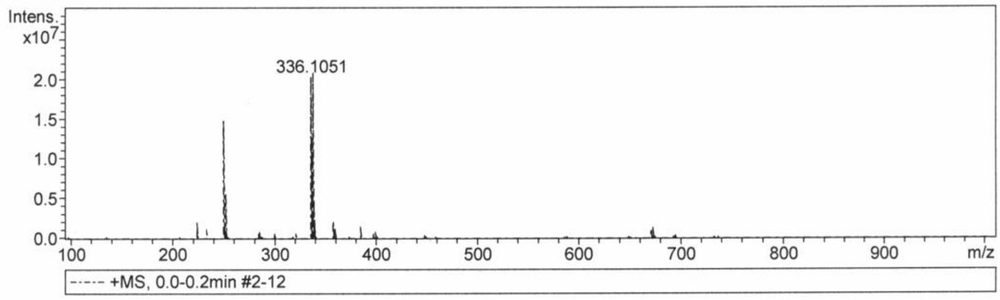

#	m/z	Res.	S/N	I	I %	FWHM
1	336.1051	13640	20367.2	20377596	100.0	0.0246
2	338.1013	27235	20329.1	20284644	99.5	0.0124

Compound **2a** ^{1}H NMR (DMSOd_6)

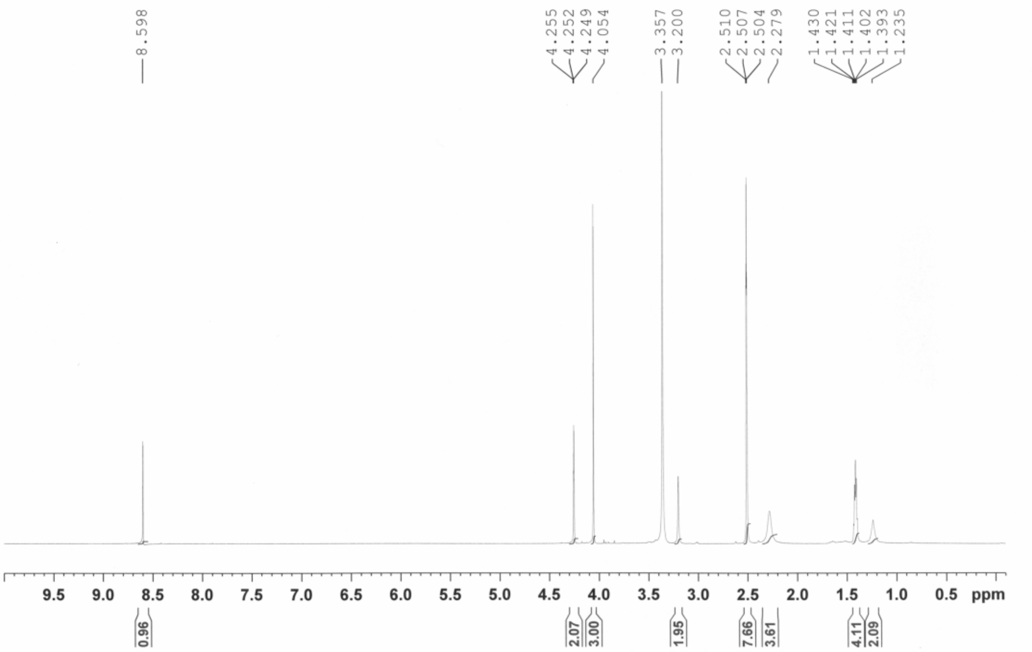

Compound **2a** ^{13}C NMR (DMSOd_6)

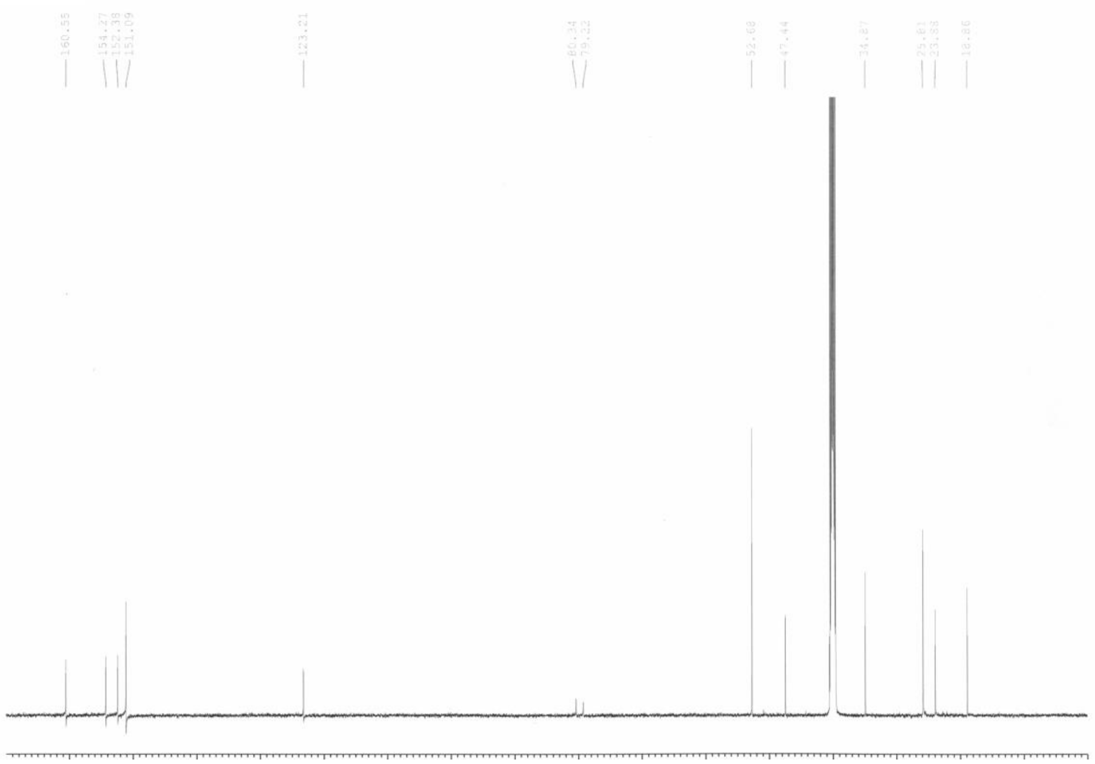

Compound **2b** HR ESIMS

Mass Spectrum List Report

Analysis Info

Analysis Name D:\Data\AK75-1.d
Method low_mass.m
Sample Name TM Low concentration
Comment

Acquisition Date 6/1/2017 1:14:44 PM

Operator KM
Instrument impact II 1825265.10082

Acquisition Parameter

Source Type	ESI	Ion Polarity	Positive	Set Nebulizer	0.3 Bar
Focus	Active	Set Capillary	4000 V	Set Dry Heater	240 °C
Scan Begin	100 m/z	Set End Plate Offset	-500 V	Set Dry Gas	4.0 l/min
Scan End	1000 m/z	Set Charging Voltage	2000 V	Set Divert Valve	Source
		Set Corona	0 nA	Set APCI Heater	0 °C

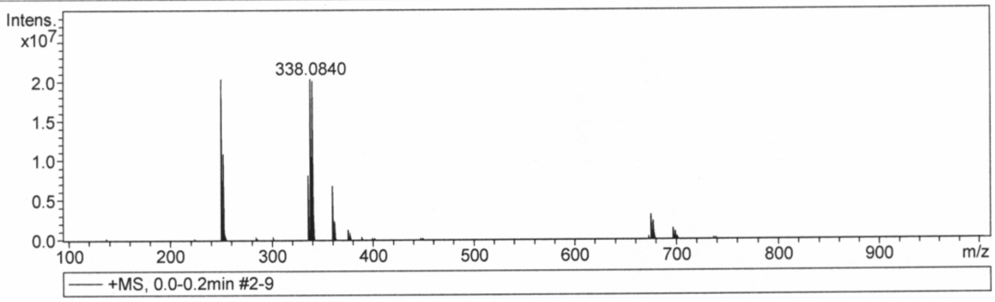

#	m/z	Res.	S/N	I	I %	FWHM
1	338.0840	13914	20197.4	20377596	100.0	0.0243
2	340.0799	30737	20025.0	20109488	98.7	0.0111
3	360.0646	43289	6647.7	6842082	33.6	0.0083

Compound **2b** ^{1}H NMR (DMSOd_6)

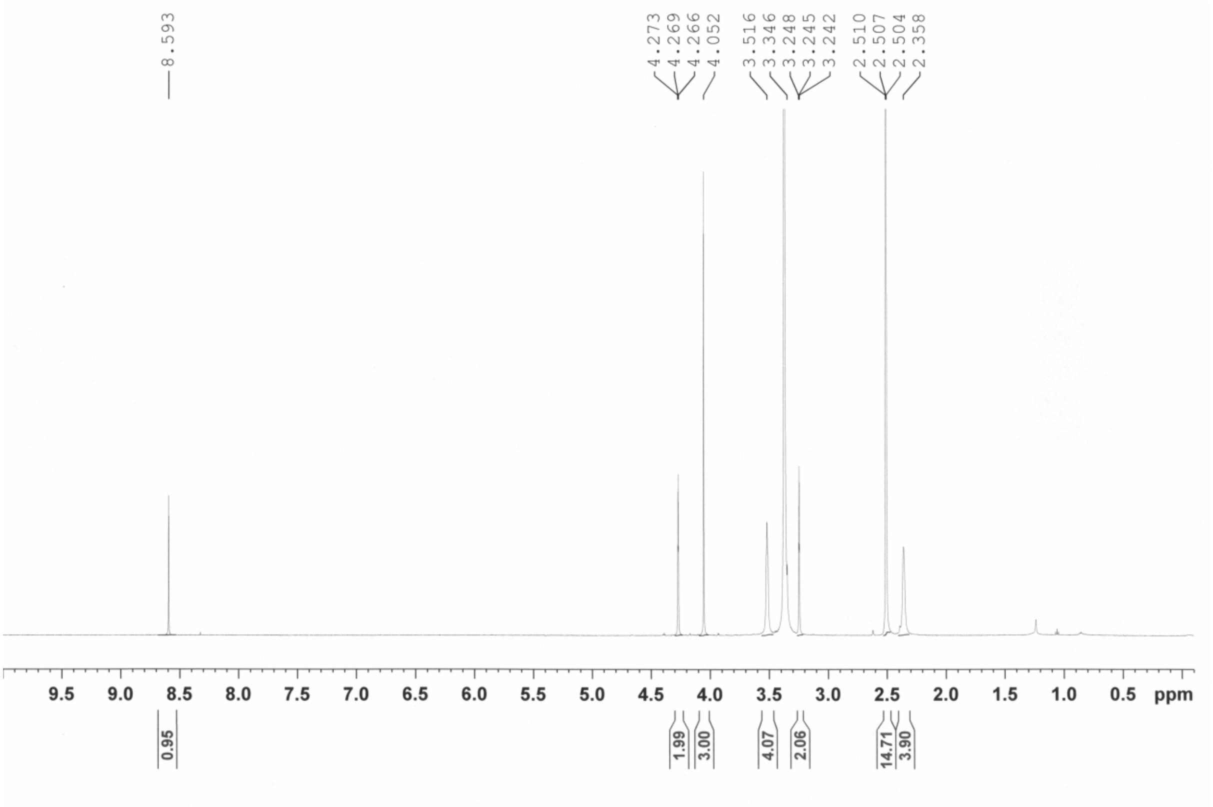

Compound **2b** ^{13}C NMR (DMSOd_6)

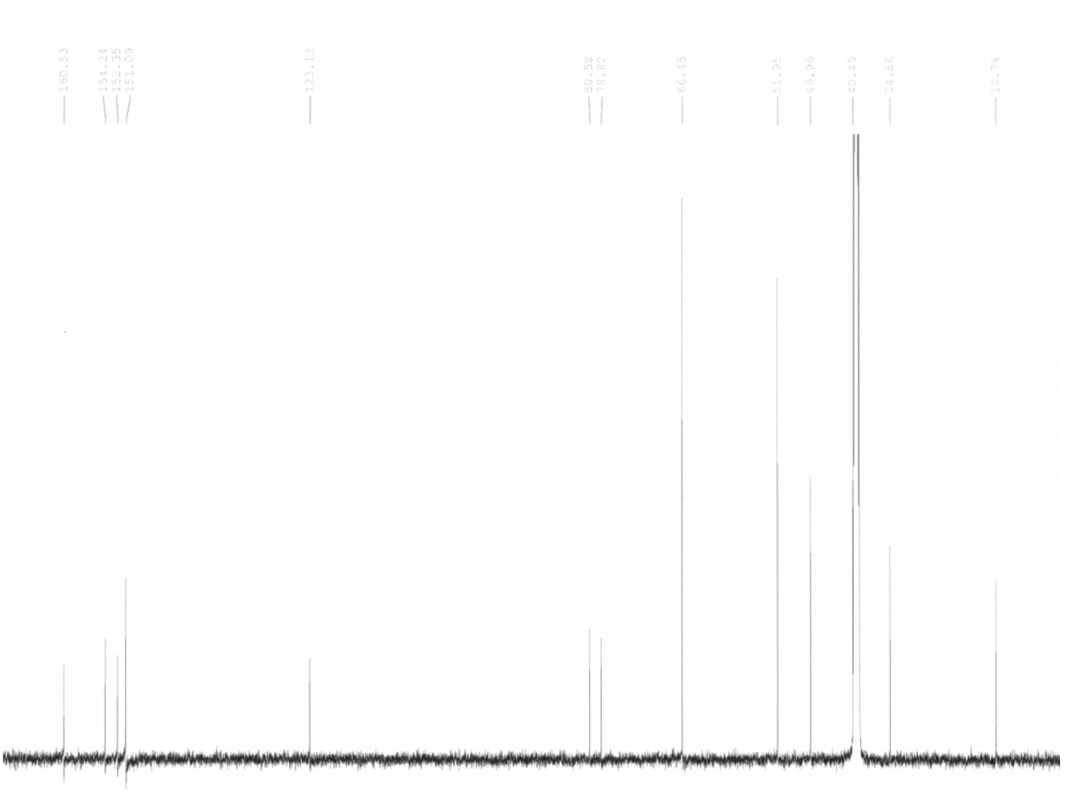

Compound **2c** HR ESIMS

Mass Spectrum List Report

Analysis Info

Acquisition Date 6/1/2017 1:24:53 PM

Analysis Name D:\Data\AK78-1.d

Method low_mass.m

Sample Name TM Low concentration

Comment

Operator KM

Instrument impact II 1825265.10082

Acquisition Parameter

Source Type	ESI	Ion Polarity	Positive	Set Nebulizer	0.3 Bar
Focus	Active	Set Capillary	4000 V	Set Dry Heater	240 °C
Scan Begin	100 m/z	Set End Plate Offset	-500 V	Set Dry Gas	4.0 l/min
Scan End	1000 m/z	Set Charging Voltage	2000 V	Set Divert Valve	Source
		Set Corona	0 nA	Set APCI Heater	0 °C

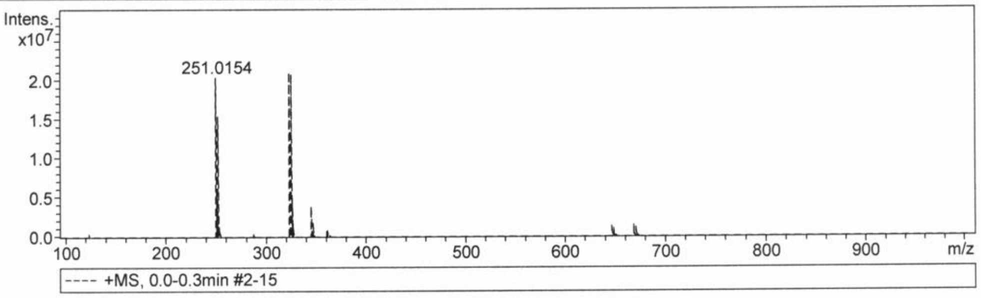

#	m/z	Res.	S/N	I	I %	FWHM
1	251.0154	16490	38264.4	20358188	99.9	0.0152
2	253.0120	35835	28792.9	15380802	75.5	0.0071
3	324.1053	12830	22863.0	20377494	100.0	0.0253
4	326.1014	23085	22829.2	20229158	99.3	0.0141

Compound **2c** ^{1}H NMR ($CDCl_3$)

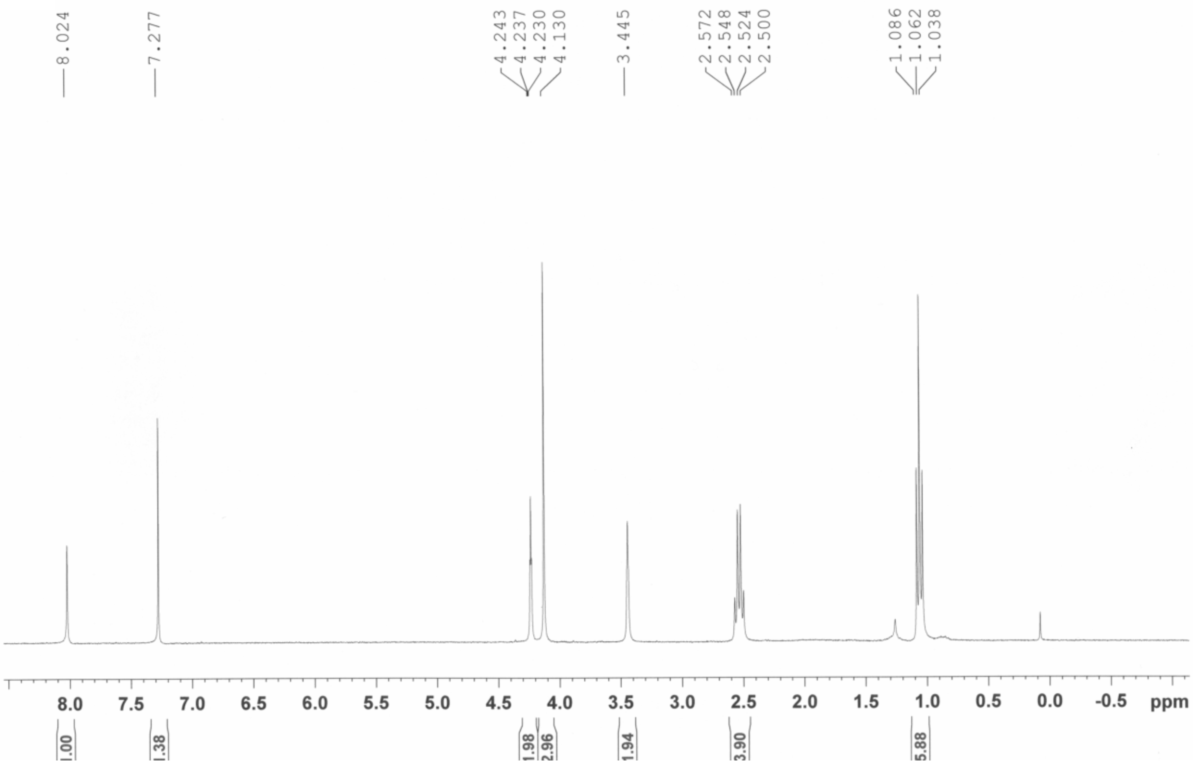

Compound **2c** ^{1}H NMR (DMSOd_6)

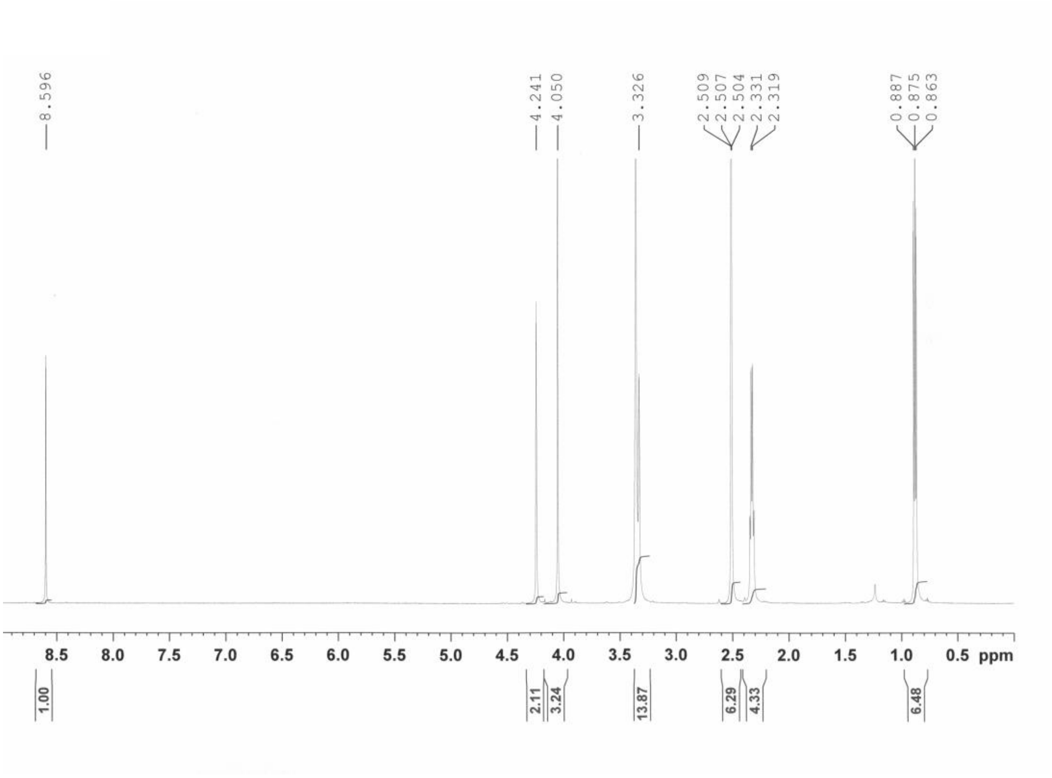

Compound **2c** ^{13}C NMR (DMSOd_6)

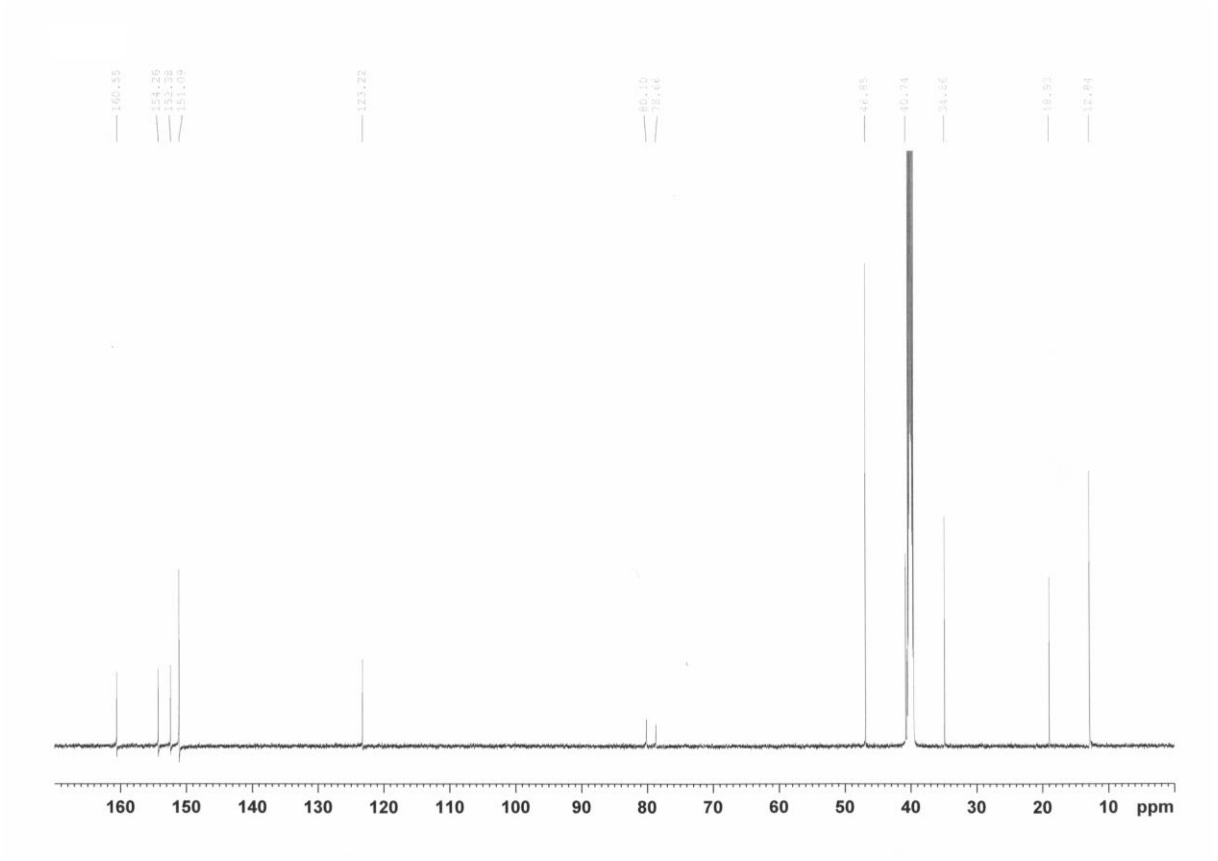

Compound **4** HR ESIMS

Compound Spectrum List Report

Analysis Info

Analysis Name	D:\Data\K79-1.d	Acquisition Date	3/10/2017 11:34:30 AM
Method	low_mass.m	Operator	KM
Sample Name	TM Low concentration	Instrument	impact II 1825265.10082
Comment			

Acquisition Parameter

Source Type	ESI	Ion Polarity	Positive	Set Nebulizer	0.3 Bar
Focus	Active	Set Capillary	4000 V	Set Dry Heater	240 °C
Scan Begin	100 m/z	Set End Plate Offset	-500 V	Set Dry Gas	4.0 l/min
Scan End	1000 m/z	Set Charging Voltage	2000 V	Set Divert Valve	Source
		Set Corona	0 nA	Set APCI Heater	0 °C

+MS, 0.0-0.2min #2-10

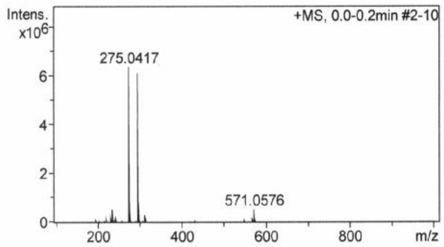

#	m/z	Res.	S/N	I	I %	FWHM
1	236.0180	29310	5384.5	540308	8.5	0.0081
2	275.0417	38114	58233.1	6338639	100.0	0.0072
3	297.0236	39906	57151.4	6087066	96.0	0.0074
4	312.9976	32347	2996.7	309829	4.9	0.0097
5	571.0576	37972	7197.0	550679	8.7	0.0150

Compound **4** ^1H NMR ($CDCl_3$)

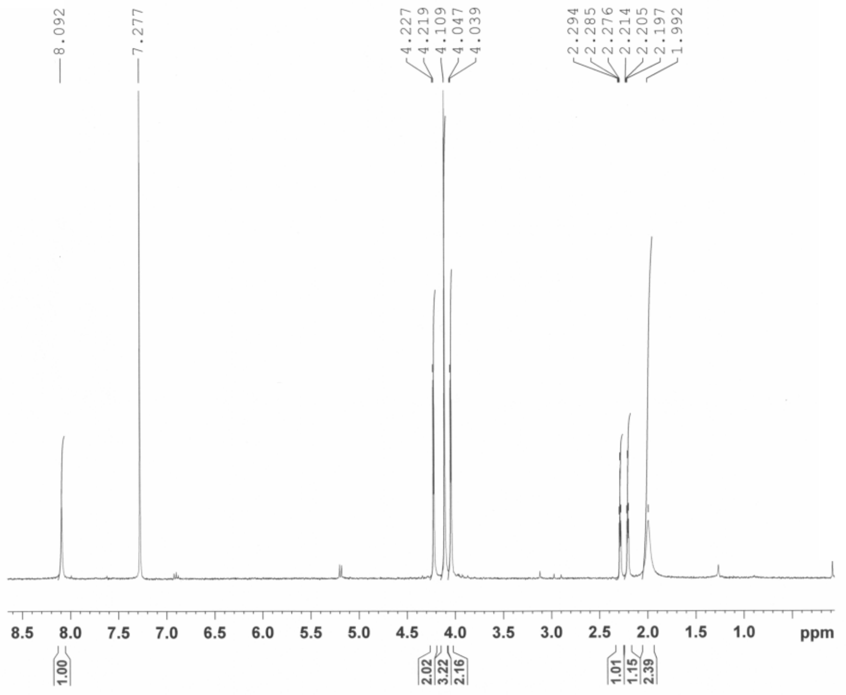

Compound **4** ^{1}H NMR (DMSOd_6)

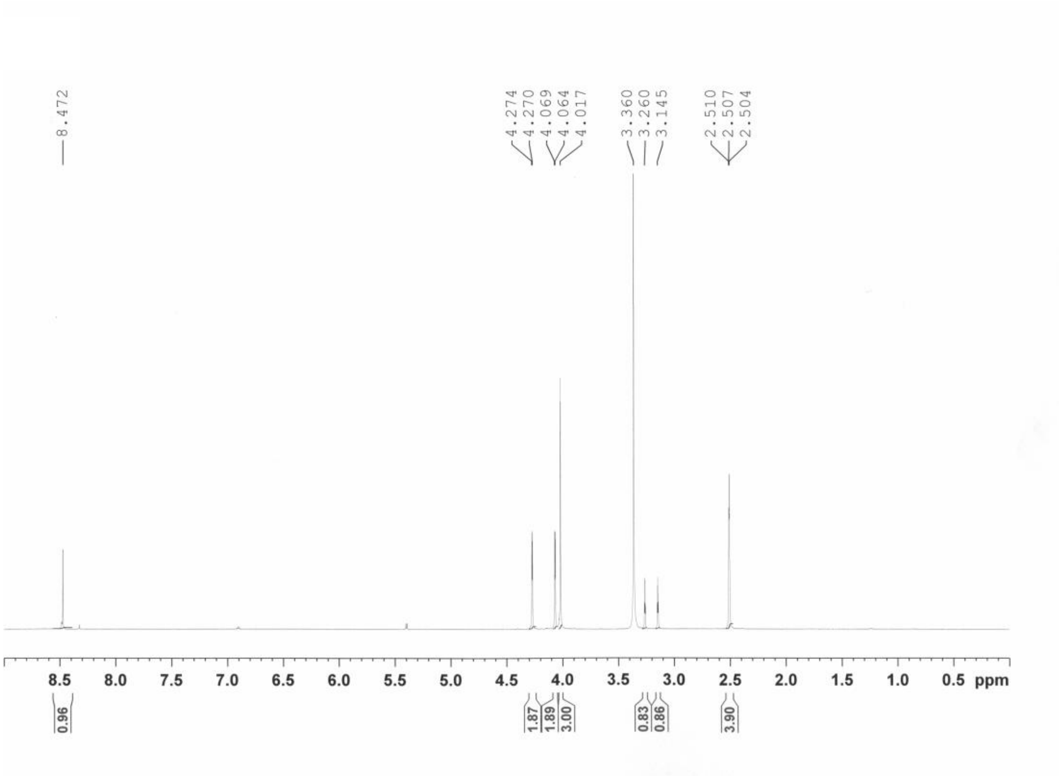

Compound **4** ^{13}C NMR (DMSOd_6)

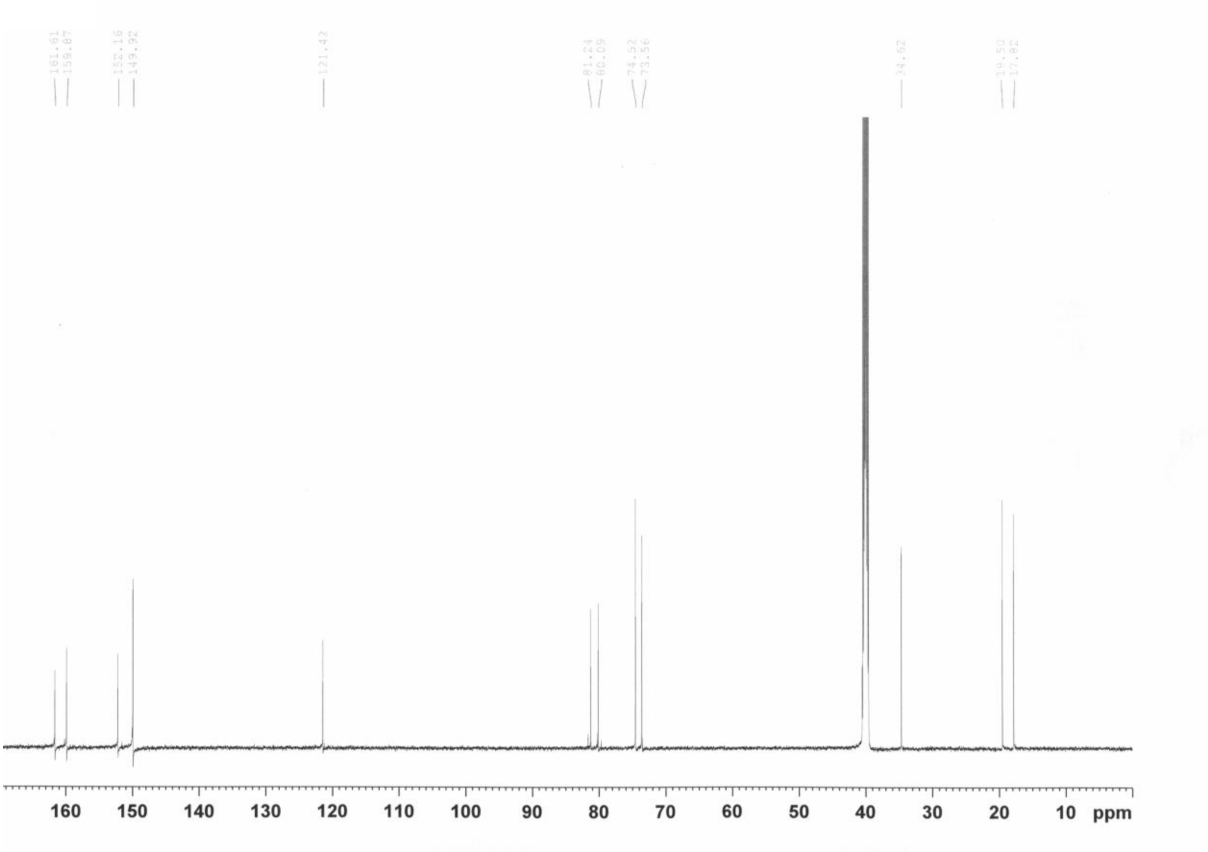

Compound **5a** HR ESIMS

Compound Spectrum List Report

Analysis Info

Acquisition Date 8/23/2016 12:44:30 PM

Analysis Name D:\Data\AK80.d
Method low_mass.m
Sample Name TM Low concentration
Comment

Operator KM
Instrument impact II 1825265.10082

Acquisition Parameter

Source Type	ESI	Ion Polarity	Positive	Set Nebulizer	0.3 Bar
Focus	Active	Set Capillary	4000 V	Set Dry Heater	240 °C
Scan Begin	100 m/z	Set End Plate Offset	-500 V	Set Dry Gas	4.0 l/min
Scan End	1000 m/z	Set Charging Voltage	2000 V	Set Divert Valve	Source
				Set APCI Heater	0 °C

+MS, 0.0-0.4min #1-22

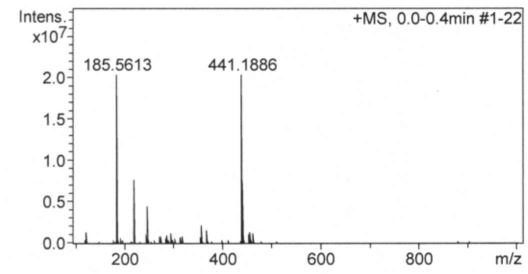

#	m/z	Res.	S/N	I	I %	FWHM
1	185.5613	20720	154515.2	20346932	99.9	0.0090
2	221.0979	34378	35335.3	7704166	37.8	0.0064
3	248.0181	35626	17476.7	4509048	22.1	0.0070
4	441.1886	26047	24170.8	20365074	100.0	0.0169

Compound **5a** ^{1}H NMR ($CDCl_3$)

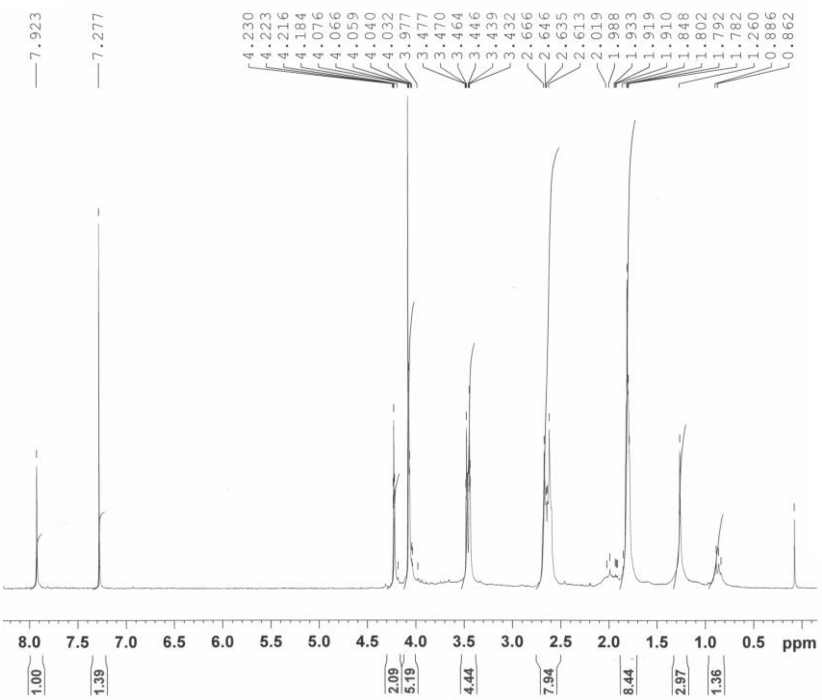

Compound **5a** ^{1}H NMR (DMSO*d*$_6$)

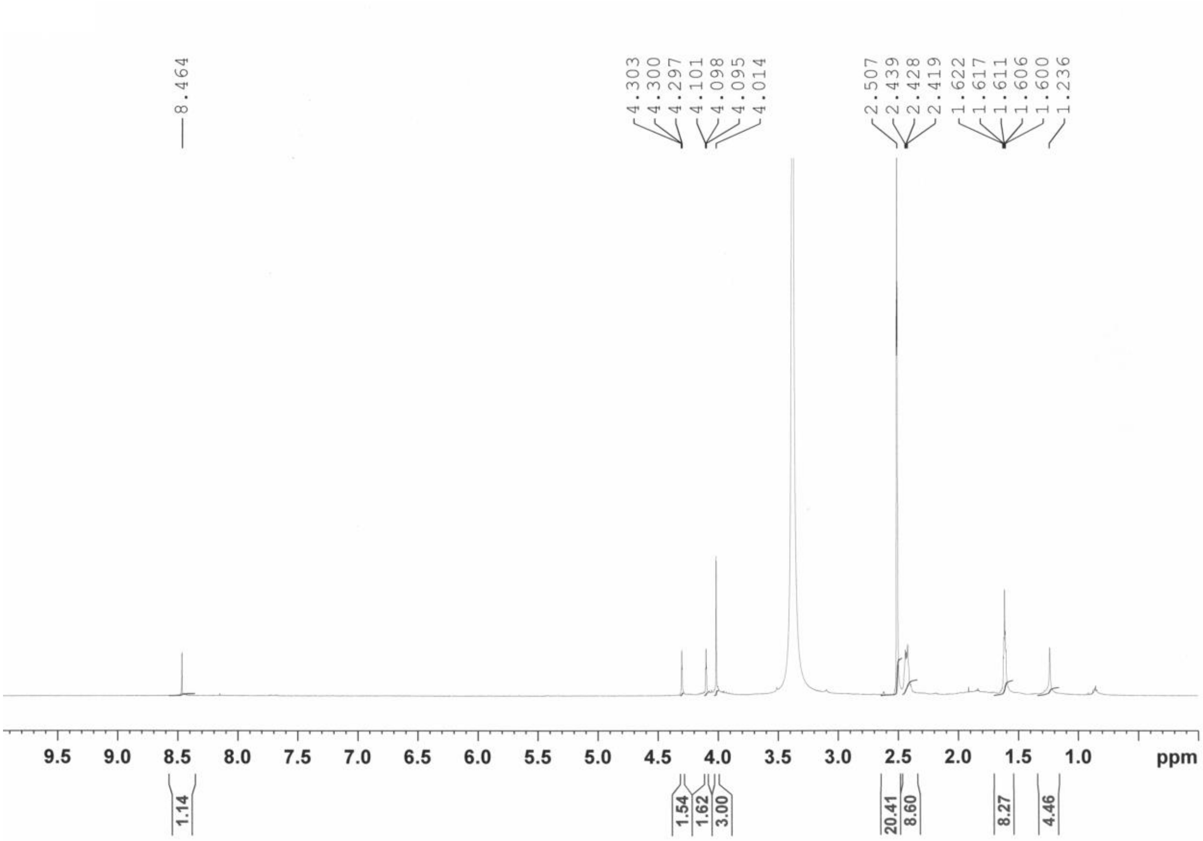

Compound **5a** ^{13}C NMR (DMSO*d*$_6$)

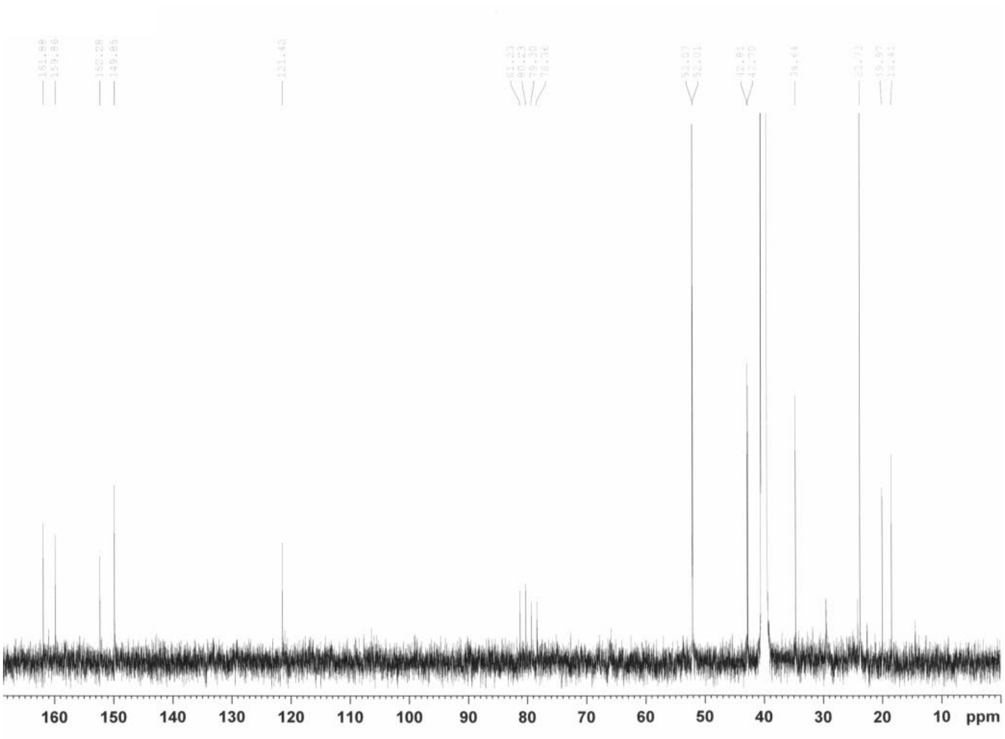

Compound **5b** HR ESIMS

Compound Spectrum List Report

Analysis Info

Analysis Name D:\Data\AK81.d
Method low_mass.m
Sample Name TM Low concentration
Comment

Acquisition Date 8/23/2016 12:47:31 PM

Operator KM
Instrument impact II 1825265.10082

Acquisition Parameter

Source Type	ESI	Ion Polarity	Positive	Set Nebulizer	0.3 Bar
Focus	Active	Set Capillary	4000 V	Set Dry Heater	240 °C
Scan Begin	100 m/z	Set End Plate Offset	-500 V	Set Dry Gas	4.0 l/min
Scan End	1000 m/z	Set Charging Voltage	2000 V	Set Divert Valve	Source
		Set Corona	0 nA	Set APCI Heater	0 °C

+MS, 0.0-0.4min #1-22

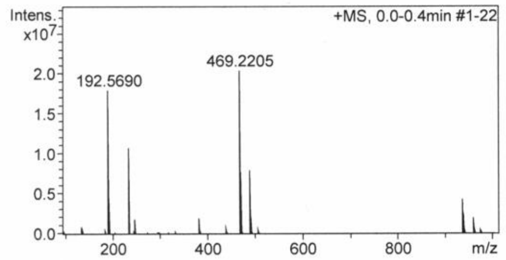

#	m/z	Res.	S/N	I	I %	FWHM
1	192.5690	30716	252741.1	17974164	88.2	0.0063
2	235.1134	35260	100560.3	10745724	52.7	0.0067
3	469.2205	13293	67355.9	20377596	100.0	0.0353
4	471.2169	45890	25341.2	7686849	37.7	0.0103
5	491.2012	49020	25480.5	7908911	38.8	0.0100

Compound **5b** ^{1}H NMR (DMSOd_6)

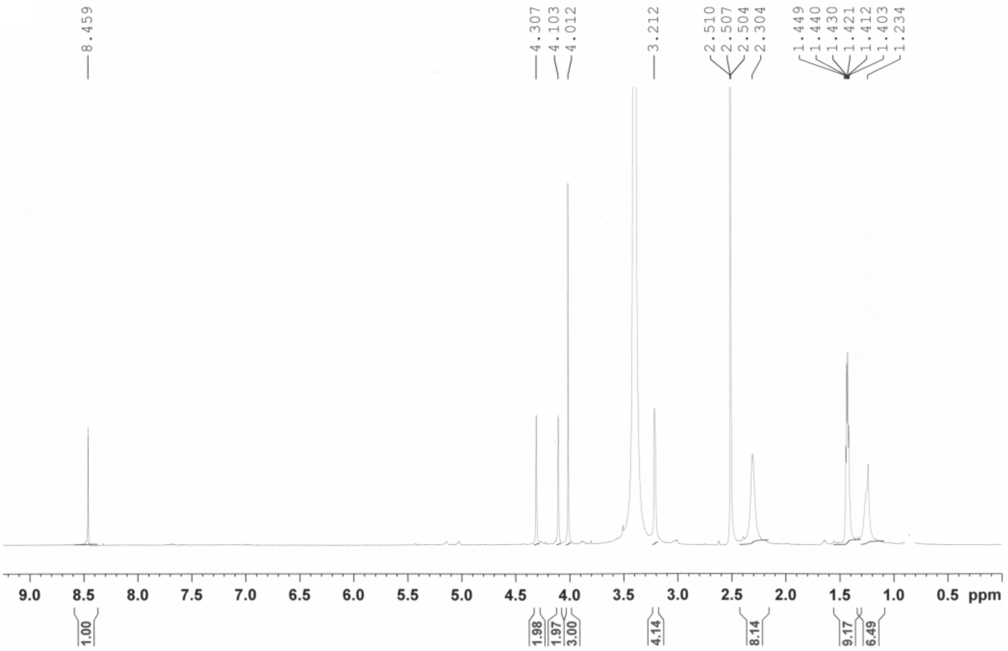

Compound **5b** ^{13}C NMR (DMSOd_6)

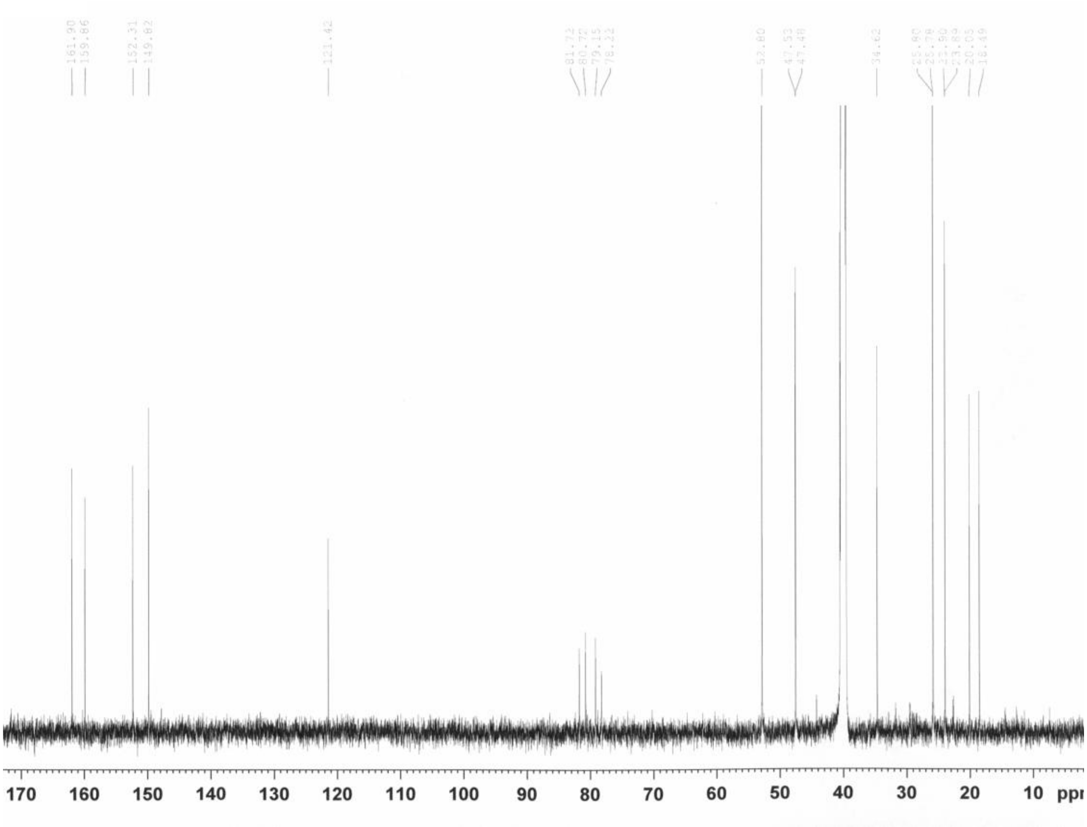

Compound **5c** HR ESIMS

Mass Spectrum List Report

Analysis Info

Analysis Name	D:\Data\AK82-1.d	Acquisition Date	6/1/2017 1:28:04 PM
Method	low_mass.m	Operator	KM
Sample Name	TM Low concentration	Instrument	impact II 1825265.10082
Comment			

Acquisition Parameter

Source Type	ESI	Ion Polarity	Positive	Set Nebulizer	0.3 Bar
Focus	Active	Set Capillary	4000 V	Set Dry Heater	240 °C
Scan Begin	100 m/z	Set End Plate Offset	-500 V	Set Dry Gas	4.0 l/min
Scan End	1000 m/z	Set Charging Voltage	2000 V	Set Divert Valve	Source
		Set Corona	0 nA	Set APCI Heater	0 °C

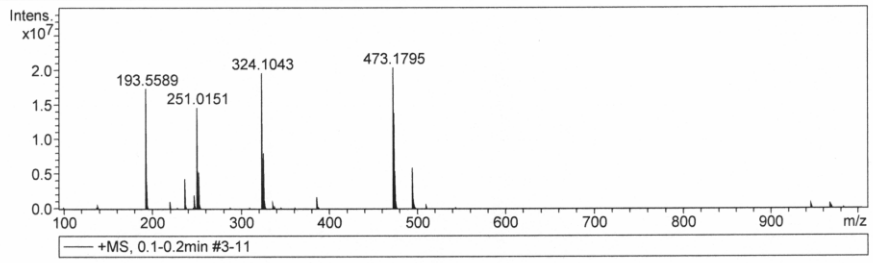

#	m/z	Res.	S/N	I	I %	FWHM
1	193.5589	31241	39598.2	17300796	84.9	0.0062
2	251.0151	36483	31874.2	14637991	71.8	0.0069
3	324.1043	32416	45359.6	19589586	96.1	0.0100
4	473.1795	16698	41096.7	20377596	100.0	0.0283
5	474.1817	48479	27783.9	13751769	67.5	0.0098

Compound **5c** ^{1}H NMR (DMSOd_6)

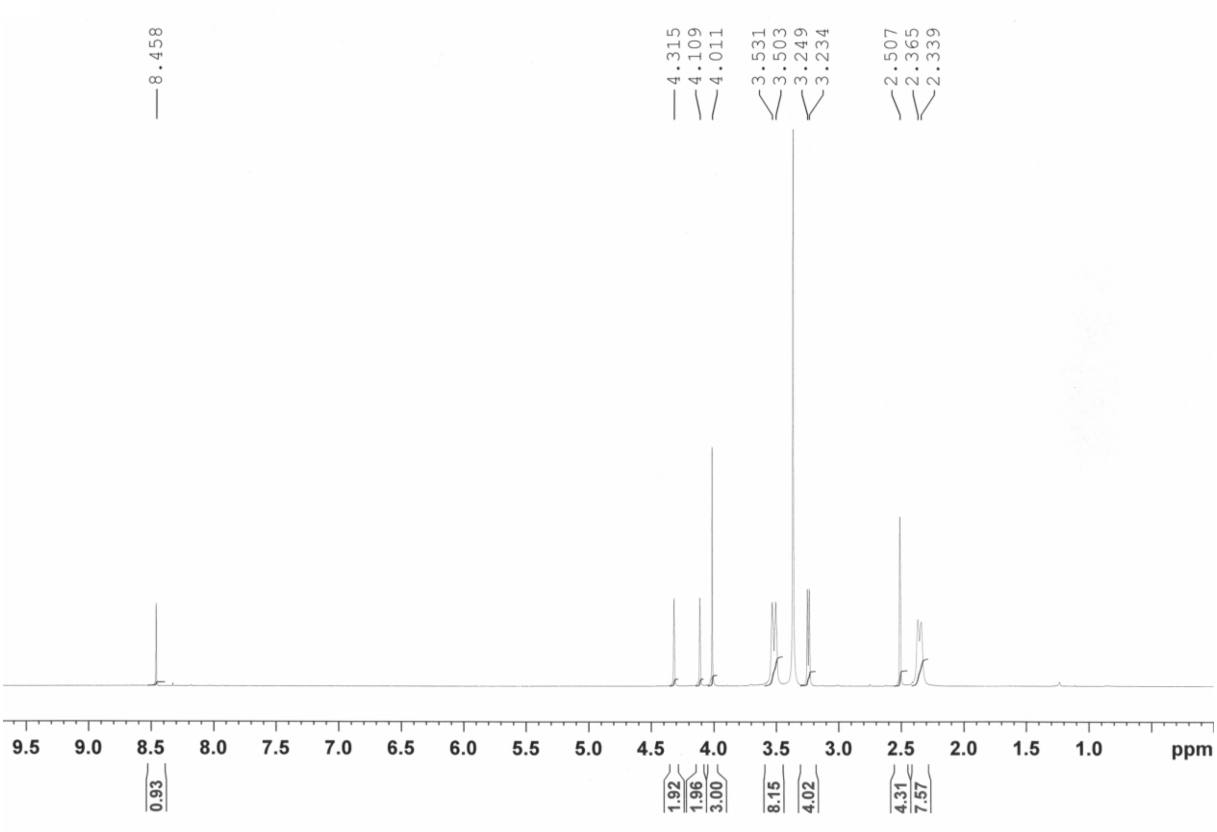

Compound **5c** ^{13}C NMR (DMSOd_6)

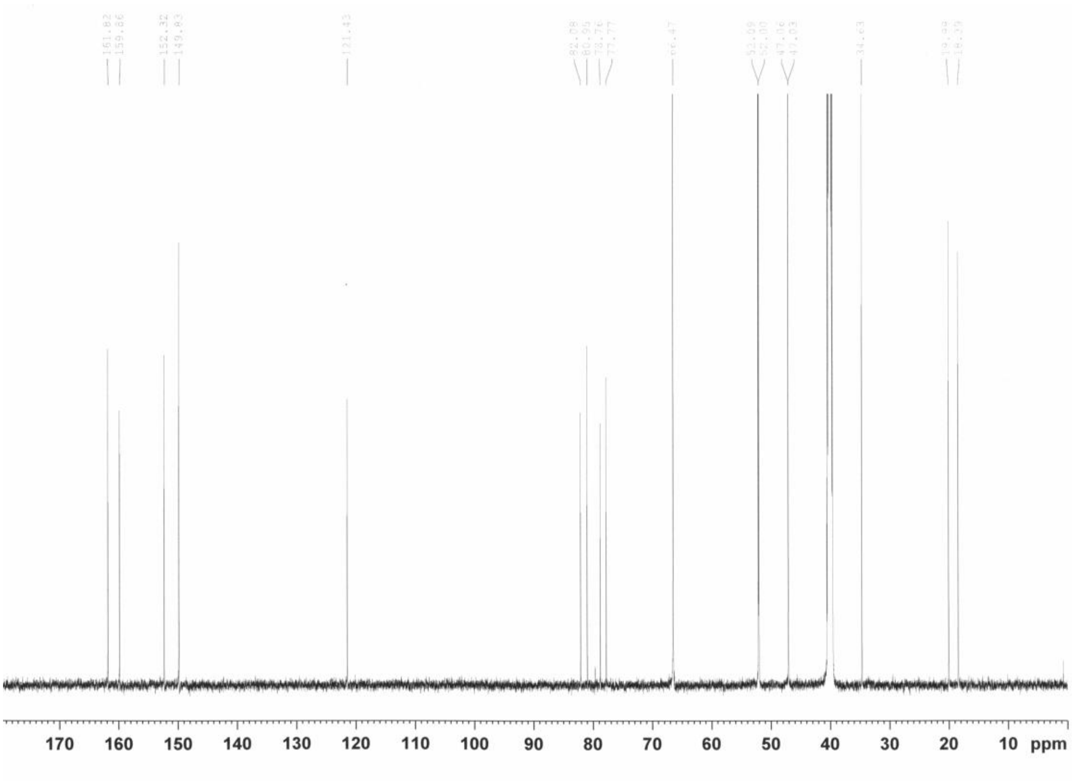

Compound **5d** HR ESIMS

Mass Spectrum List Report

Analysis Info

Acquisition Date 6/1/2017 1:36:21 PM

Analysis Name D:\Data\AK83-2.d
Method low_mass.m
Sample Name TM Low concentration
Comment

Operator KM
Instrument impact II 1825265.10082

Acquisition Parameter

Source Type	ESI	Ion Polarity	Positive	Set Nebulizer	0.3 Bar
Focus	Active	Set Capillary	4000 V	Set Dry Heater	240 °C
Scan Begin	100 m/z	Set End Plate Offset	-500 V	Set Dry Gas	4.0 l/min
Scan End	1000 m/z	Set Charging Voltage	2000 V	Set Divert Valve	Source
		Set Corona	0 nA	Set APCI Heater	0 °C

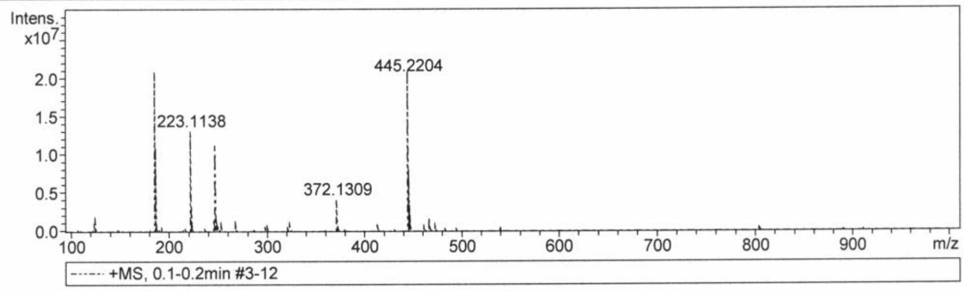

#	m/z	Res.	S/N	I	I %	FWHM
1	223.1138	34394	34899.3	13180236	64.7	0.0065
2	248.0183	36799	26165.8	10760982	52.8	0.0067
3	372.1309	42947	9135.2	4258956	20.9	0.0087
4	445.2204	23784	34718.7	20364126	100.0	0.0187

Compound **5d** ^{1}H NMR ($CDCl_3$)

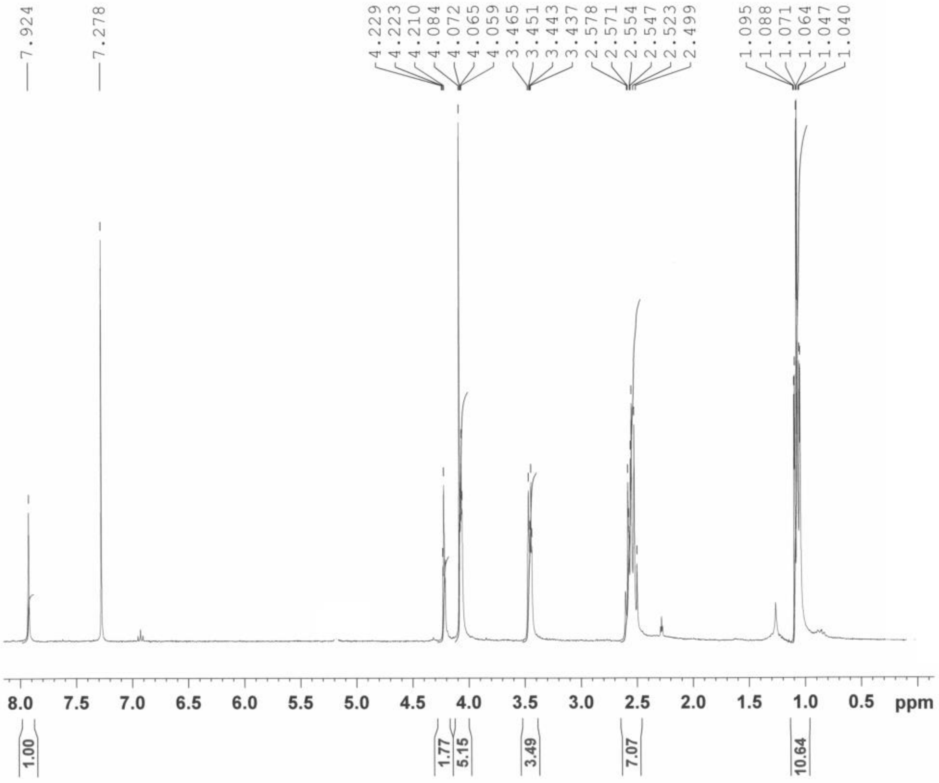

Compound **5d** ^{13}C NMR (DMSOd_6)

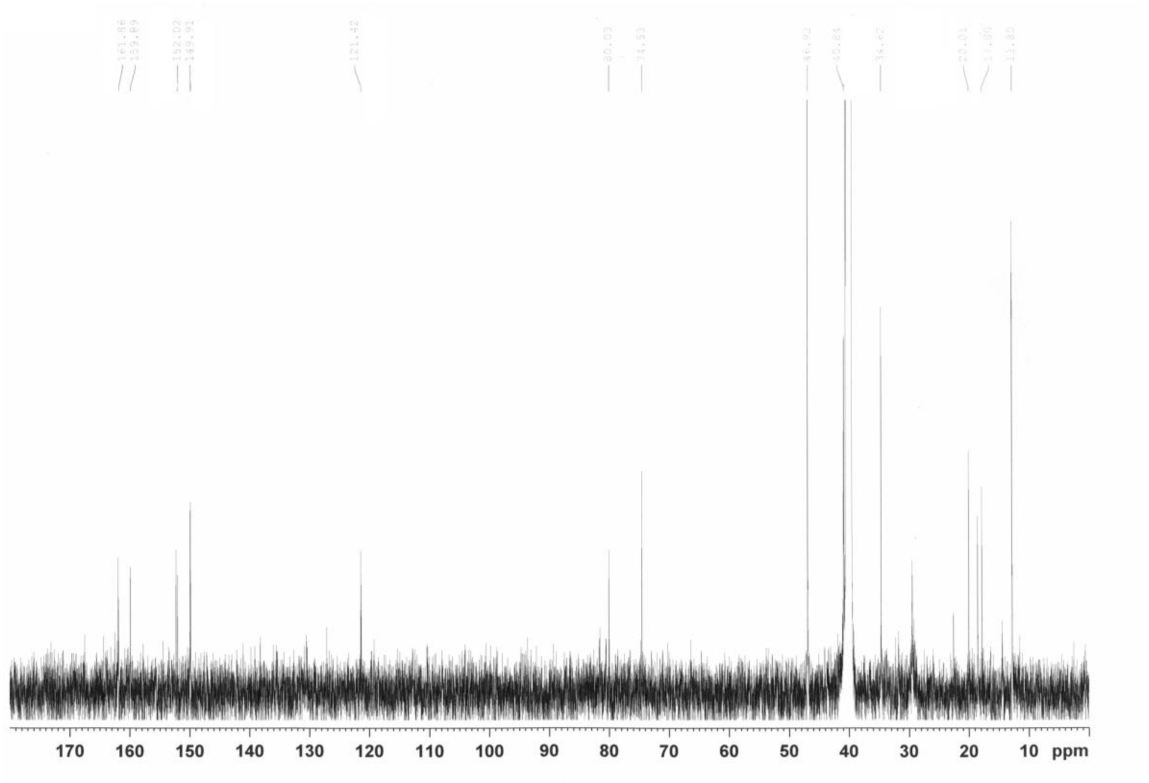

Compound **9** HR ESIMS

Mass Spectrum List Report

Analysis Info

Analysis Name	D:\Data\AK68-1.d	Acquisition Date	6/1/2017 1:44:35 PM
Method	low_mass.m	Operator	KM
Sample Name	TM Low concentration	Instrument	impact II 1825265.10082
Comment			

Acquisition Parameter

Source Type	ESI	Ion Polarity	Positive	Set Nebulizer	0.3 Bar
Focus	Active	Set Capillary	4000 V	Set Dry Heater	240 °C
Scan Begin	100 m/z	Set End Plate Offset	-500 V	Set Dry Gas	4.0 l/min
Scan End	1000 m/z	Set Charging Voltage	2000 V	Set Divert Valve	Source
		Set Corona	0 nA	Set APCI Heater	0 °C

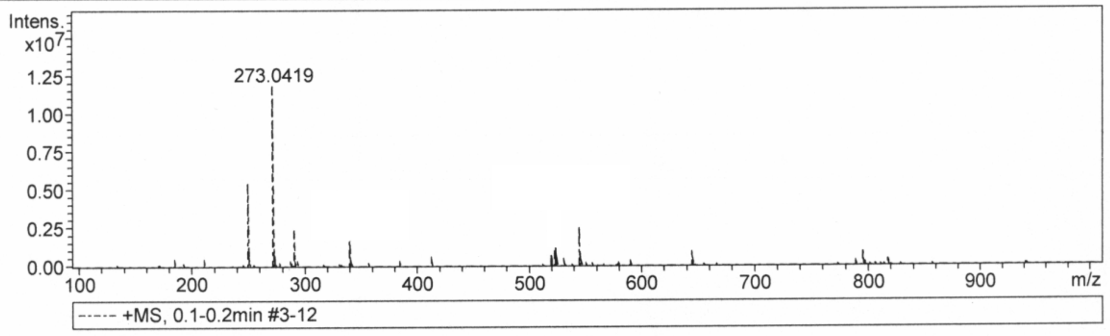

#	m/z	Res.	S/N	I	I %	FWHM
1	251.0600	36362	28252.7	5163503	43.8	0.0069
2	273.0419	37639	58345.6	11796161	100.0	0.0073

Compound **9** ^{1}HNMR(DMSOd_6)

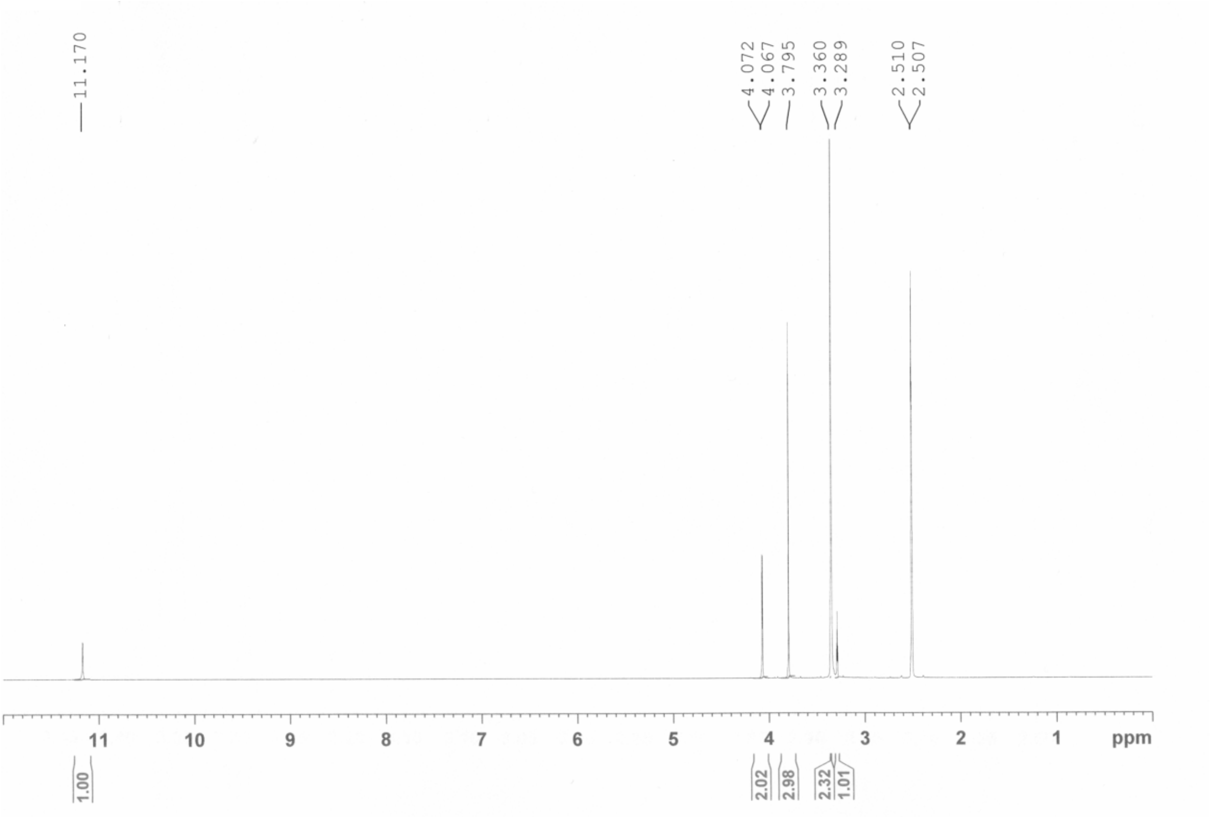

Compound **9** ^{13}C NMR (DMSOd_6)

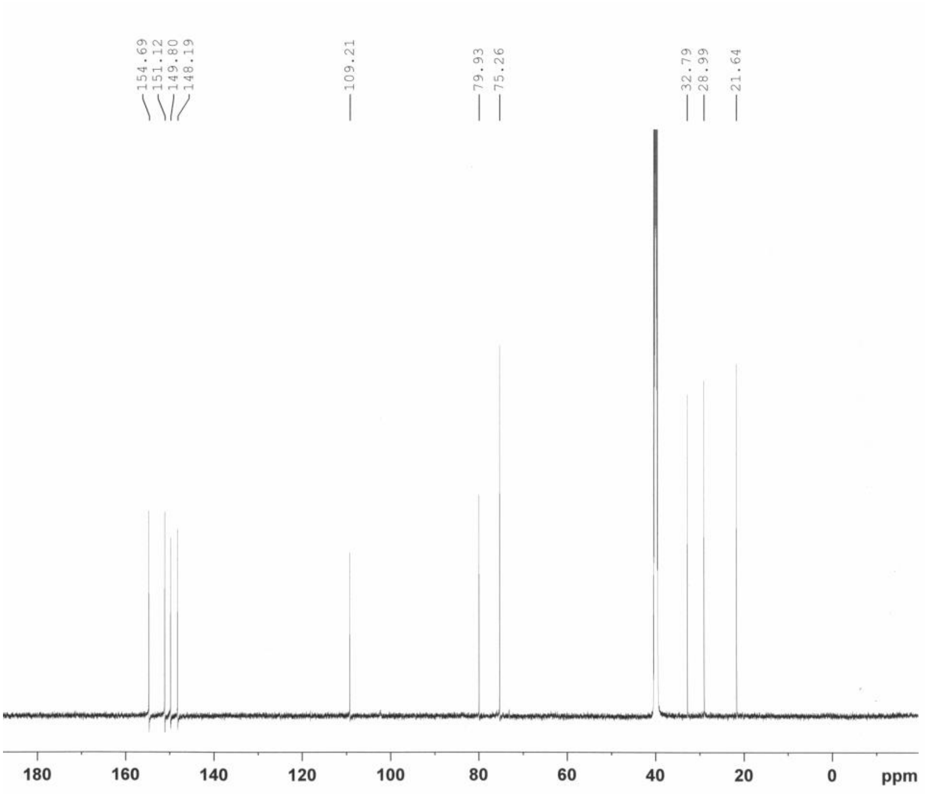

Compound **10a** HR ESIMS

Compound Spectrum List Report

Analysis Info

Acquisition Date 8/23/2016 12:04:58 PM

Analysis Name D:\Data\AK86.d

Method low_mass.m

Sample Name TM Low concentration

Comment

Operator KM

Instrument impact II 1825265.10082

Acquisition Parameter

Source Type	ESI	Ion Polarity	Positive	Set Nebulizer	0.3 Bar
Focus	Active	Set Capillary	4000 V	Set Dry Heater	240 °C
Scan Begin	100 m/z	Set End Plate Offset	-500 V	Set Dry Gas	4.0 l/min
Scan End	1000 m/z	Set Charging Voltage	2000 V	Set Divert Valve	Source
		Set Corona	0 nA	Set APCI Heater	0 °C

+MS, 0.0-0.4min #1-22

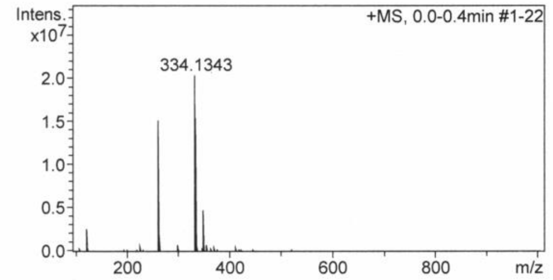

#	m/z	Res.	S/N	I	I %	FWHM
1	263.0597	37447	115005.2	15146227	74.3	0.0070
2	334.1343	11405	89092.5	20377596	100.0	0.0293
3	335.1361	39756	66800.8	15366674	75.4	0.0084
4	350.1279	41627	19039.5	4769081	23.4	0.0084

Compound **10a** ^{1}H NMR (DMSOd_6)

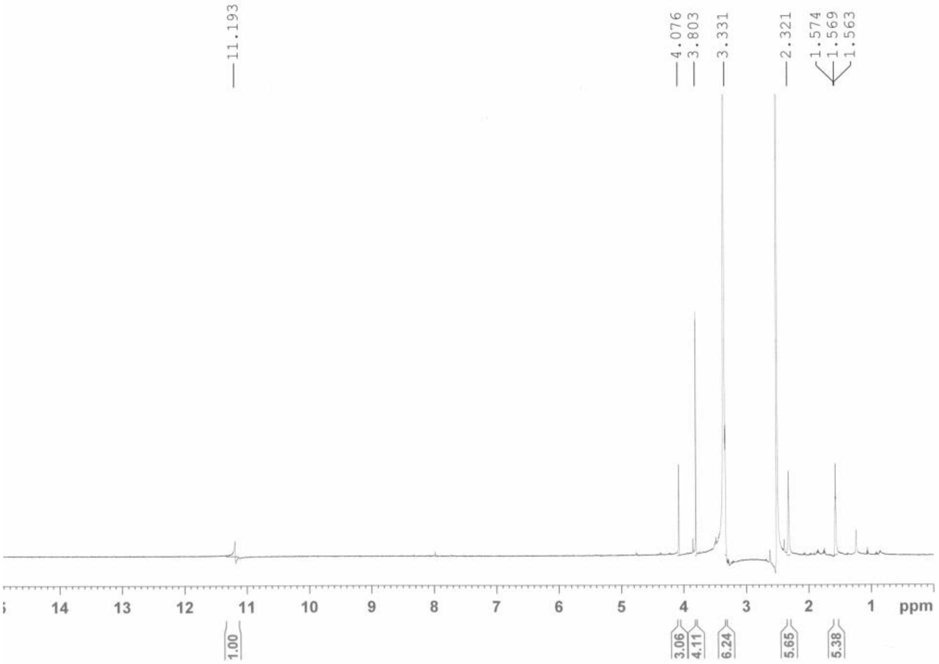

Compound **10a** ^{13}C NMR (DMSOd_6)

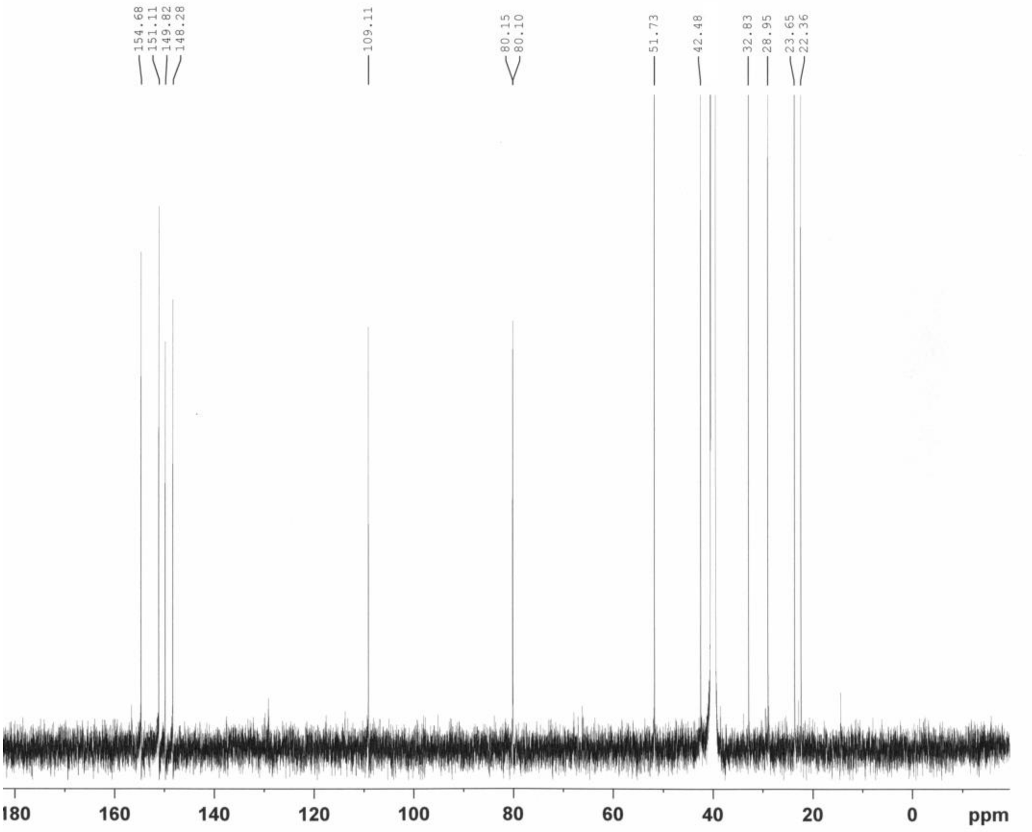

Compound **10b** HR ESIMS

Compound Spectrum List Report

Analysis Info

Analysis Name	D:\Data\ak72a.d	Acquisition Date	7/1/2016 10:07:21 AM
Method	low_mass.m	Operator	KM
Sample Name	TM Low concentration	Instrument	impact II 1825265.10082
Comment			

Acquisition Parameter

Source Type	ESI	Ion Polarity	Positive	Set Nebulizer	0.5 Bar
Focus	Active	Set Capillary	4000 V	Set Dry Heater	240 °C
Scan Begin	100 m/z	Set End Plate Offset	-500 V	Set Dry Gas	4.5 l/min
Scan End	1000 m/z	Set Charging Voltage	2000 V	Set Divert Valve	Source
		Set Corona	0 nA	Set APCI Heater	0 °C

+MS, 0.0-0.4min #1-25

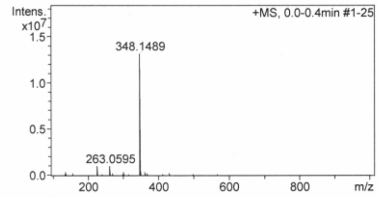

#	m/z	Res.	S/N	I	I %	FWHM
1	226.9513	31139	26865.4	1030932	7.8	0.0073
2	263.0595	32607	19414.1	1091357	8.3	0.0081
3	302.0742	27579	3101.8	244945	1.9	0.0110
4	348.1489	42052	112591.0	13175050	100.0	0.0083
5	362.9261	29022	1910.5	240472	1.8	0.0125

Compound **10b** ^{1}H NMR (DMSOd_6)

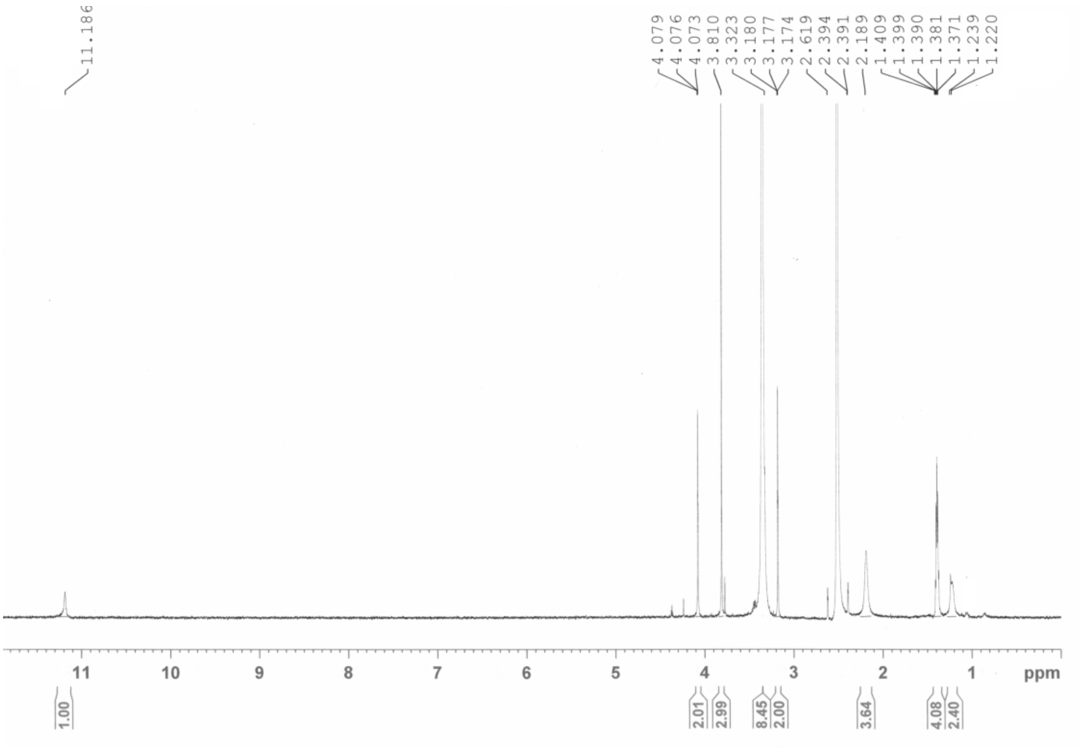

Compound **10b** ^{13}C NMR (DMSOd_6)

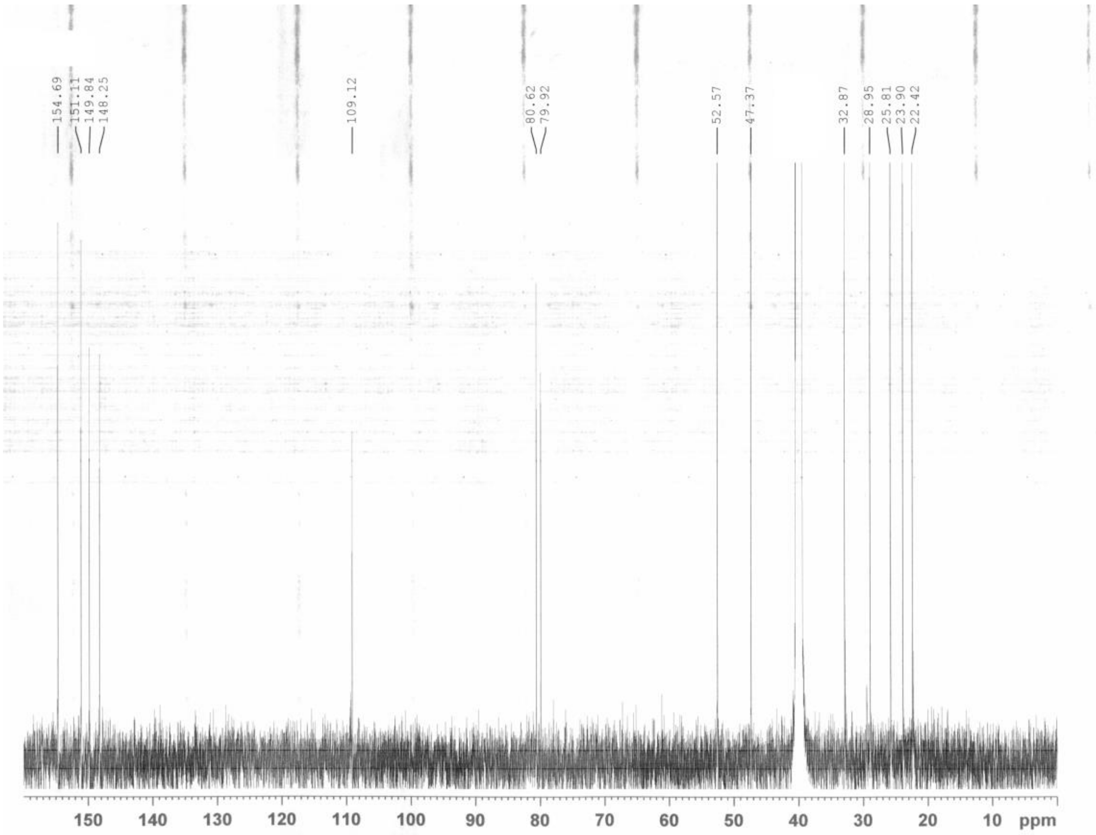

Compound **10c** HR ESIMS

Compound Spectrum List Report

Analysis Info

Analysis Name	D:\Data\AK73-1.d	Acquisition Date	6/21/2017 9:11:59 AM
Method	low_mass.m	Operator	KM
Sample Name	TM Low concentration	Instrument	impact II 1825265.10082
Comment			

Acquisition Parameter

Source Type	ESI	Ion Polarity	Positive	Set Nebulizer	0.3 Bar
Focus	Active	Set Capillary	4000 V	Set Dry Heater	240 °C
Scan Begin	100 m/z	Set End Plate Offset	-500 V	Set Dry Gas	4.0 l/min
Scan End	1000 m/z	Set Charging Voltage	2000 V	Set Divert Valve	Source
		Set Corona	0 nA	Set APCI Heater	0 °C

+MS, 0.3-0.5min #17-27

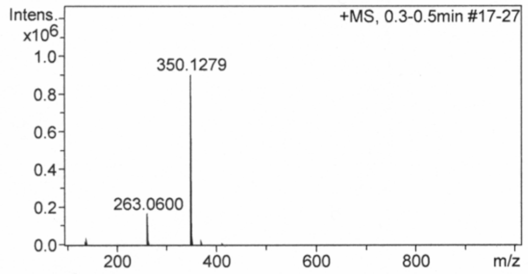

#	m/z	Res.	S/N	I	I %	FWHM
1	263.0600	22867	5220.0	168208	18.6	0.0115
2	350.1279	29609	28799.5	902711	100.0	0.0118

Compound **10c** ^{1}H NMR (DMSOd_6)

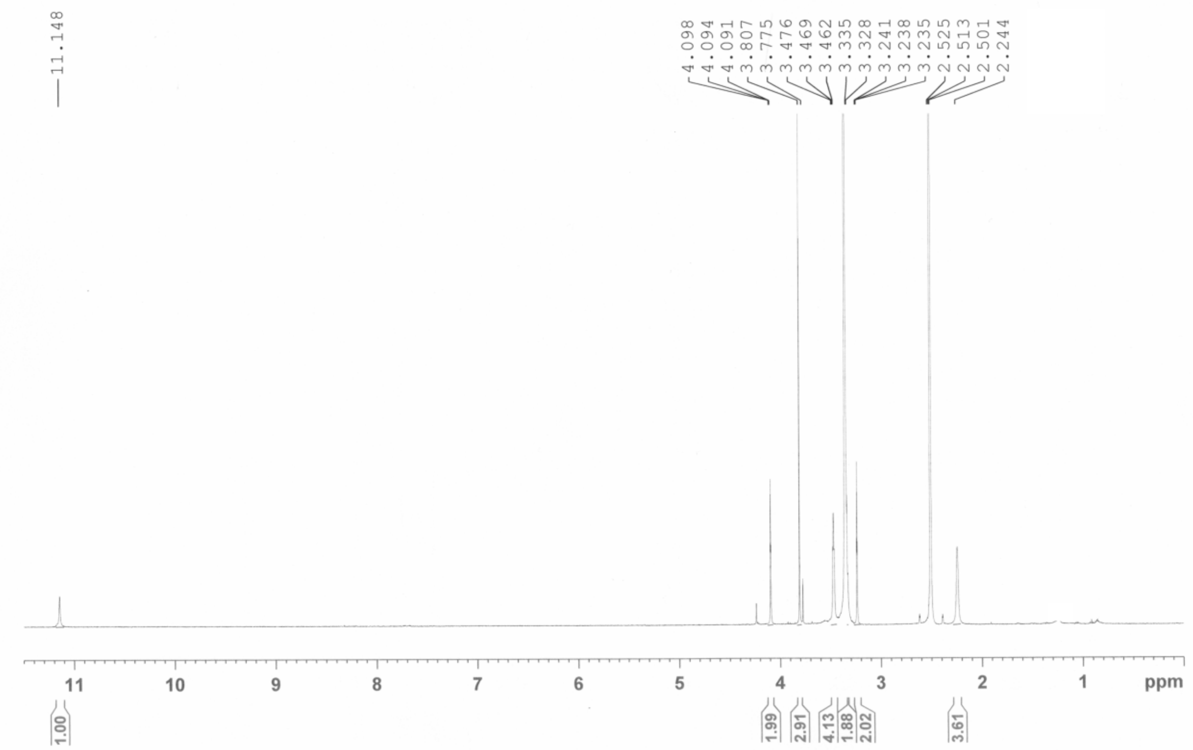

Compound **10c** ^{13}C NMR (DMSOd_6)

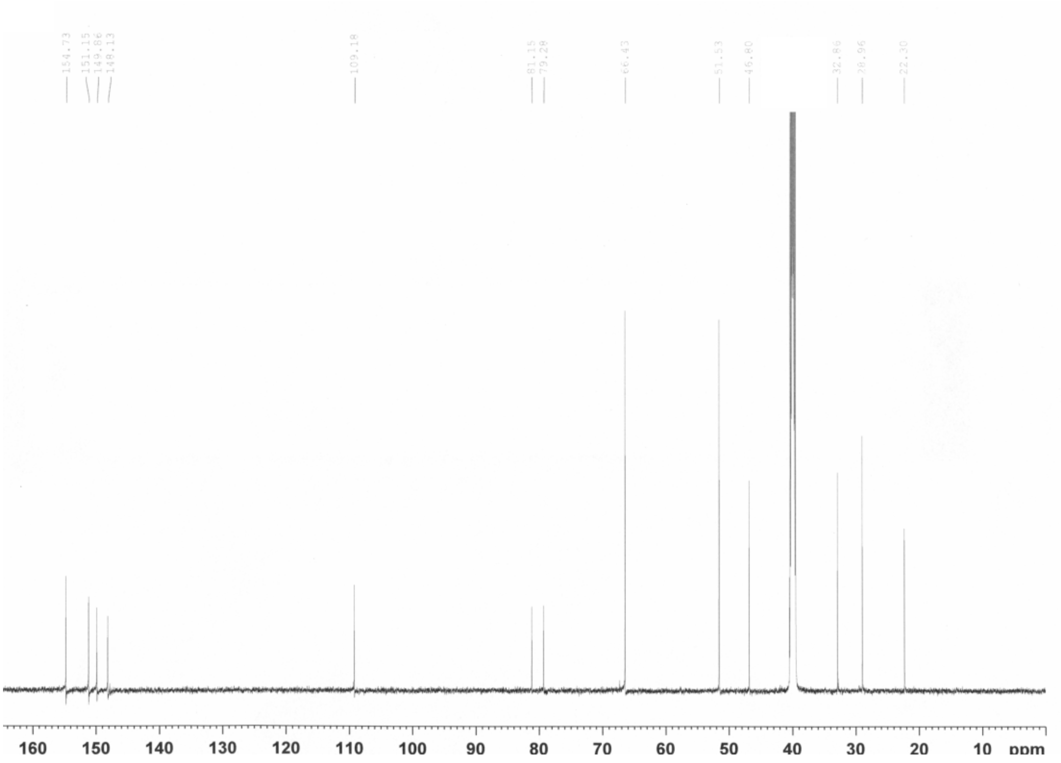

Compound **10d** HR ESIMS

Compound Spectrum List Report

Analysis Info

Acquisition Date 3/10/2017 11:40:24 AM

Analysis Name D:\Data\K74-1.d

Method low_mass.m

Sample Name TM Low concentration

Comment

Operator KM

Instrument impact II 1825265.10082

Acquisition Parameter

Source Type	ESI	Ion Polarity	Positive	Set Nebulizer	0.3 Bar
Focus	Active	Set Capillary	4000 V	Set Dry Heater	240 °C
Scan Begin	100 m/z	Set End Plate Offset	-500 V	Set Dry Gas	4.0 l/min
Scan End	1000 m/z	Set Charging Voltage	2000 V	Set Divert Valve	Source
		Set Corona	0 nA	Set APCI Heater	0 °C

+MS, 0.0-0.1min #2-7

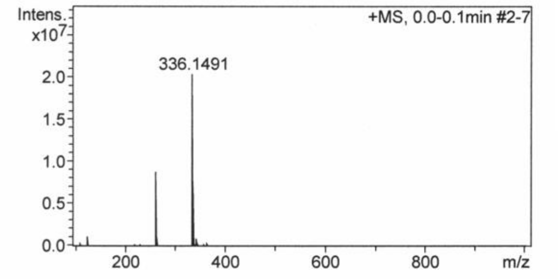

#	m/z	Res.	S/N	I	I %	FWHM
1	263.0596	37600	33402.7	8764769	43.0	0.0070
2	336.1491	19304	51619.6	20377596	100.0	0.0174
3	337.1517	41398	17933.7	7064238	34.7	0.0081

Compound **10d** ^{1}H NMR (DMSOd_6)

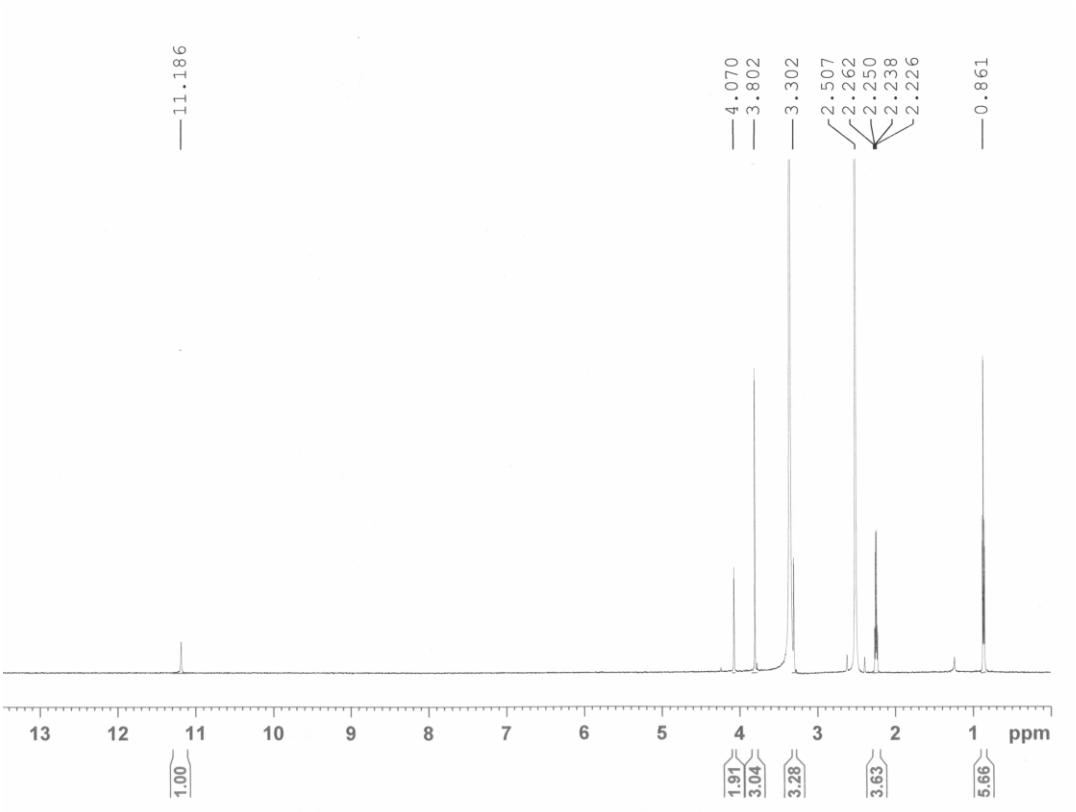

Compound **10d** ^{13}C NMR (DMSOd_6)

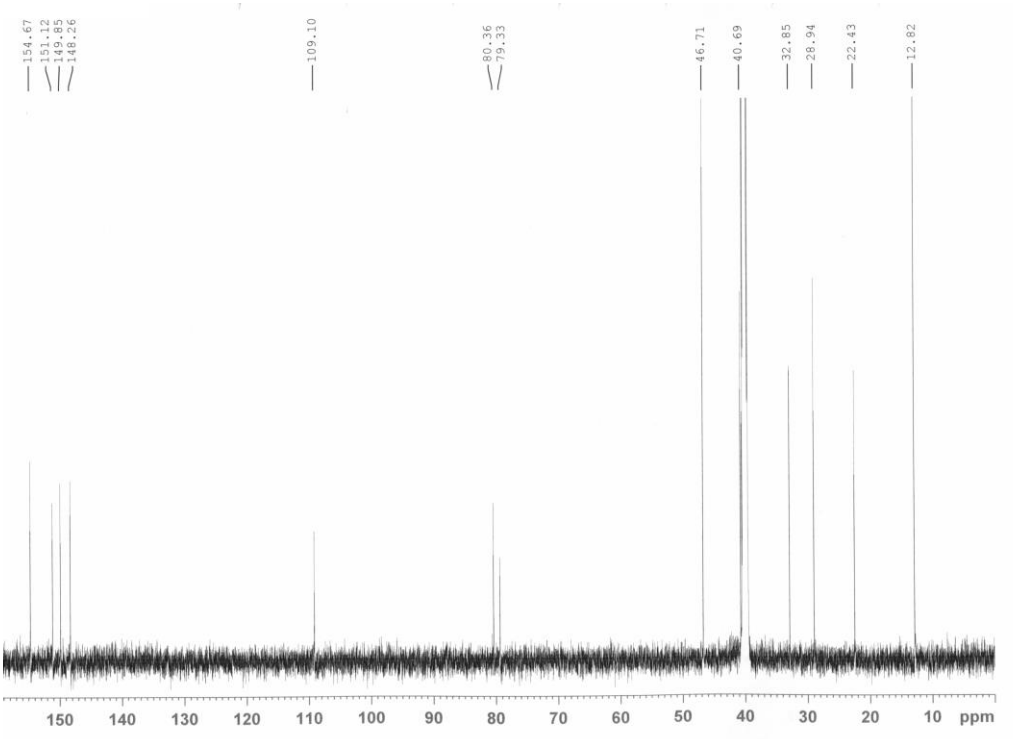

Compound **14** HR ESIMS

Compound Spectrum List Report

Analysis Info

Analysis Name D:\Data\AK89-1.d
Method low_mass.m
Sample Name TM Low concentration
Comment

Acquisition Date 2/15/2017 10:51:42 AM
Operator KM
Instrument impact II 1825265.10082

Acquisition Parameter

Source Type	ESI	Ion Polarity	Positive	Set Nebulizer	0.3 Bar
Focus	Active	Set Capillary	4000 V	Set Dry Heater	240 °C
Scan Begin	100 m/z	Set End Plate Offset	-500 V	Set Dry Gas	4.0 l/min
Scan End	1000 m/z	Set Charging Voltage	2000 V	Set Divert Valve	Source
		Set Corona	0 nA	Set APCI Heater	0 °C

+MS, 0.1-0.3min #3-19

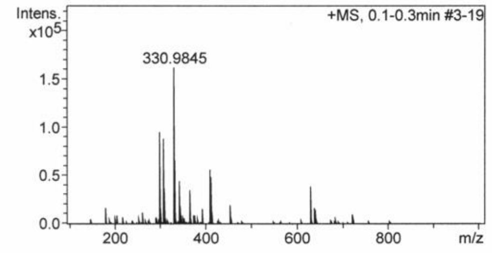

#	m/z	Res.	S/N	I	I %	FWHM
1	301.1408	23225	3274.3	94830	58.5	0.0130
2	309.0027	23456	2974.7	88383	54.5	0.0132
3	330.9845	24580	5144.5	162142	100.0	0.0135

Compound **14** ^{1}H NMR (DMSOd_6)

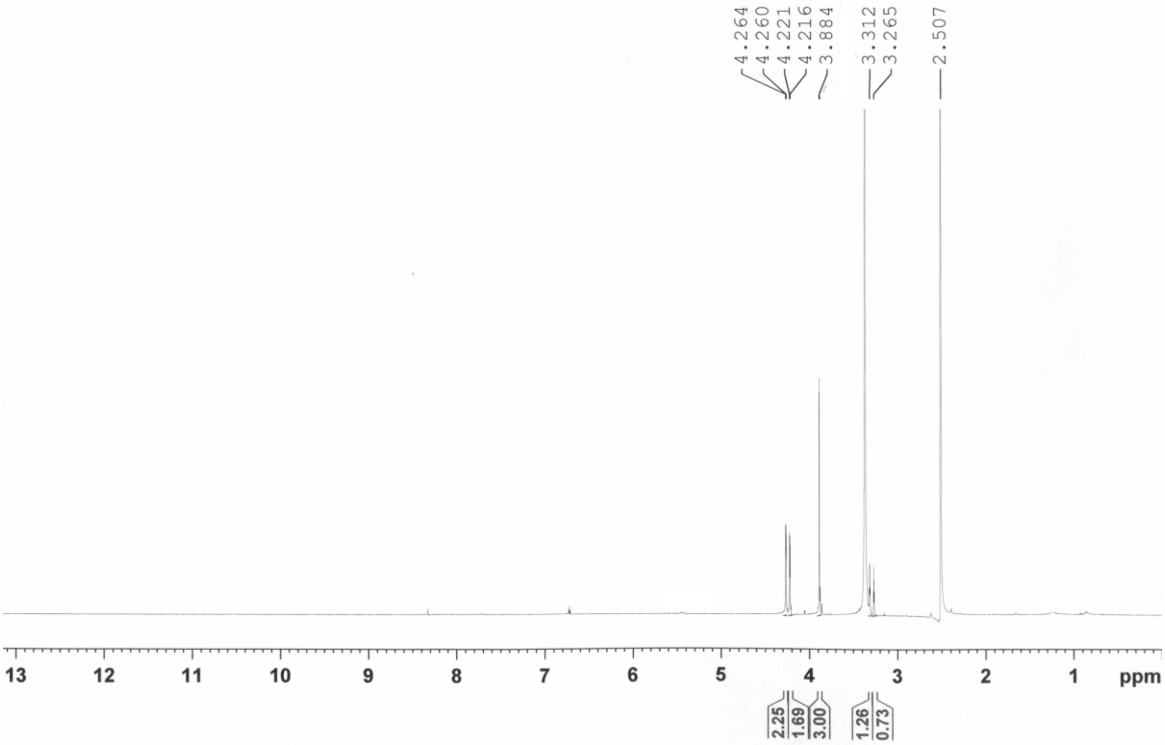

Compound **14** ^{13}C NMR (DMSOd_6)

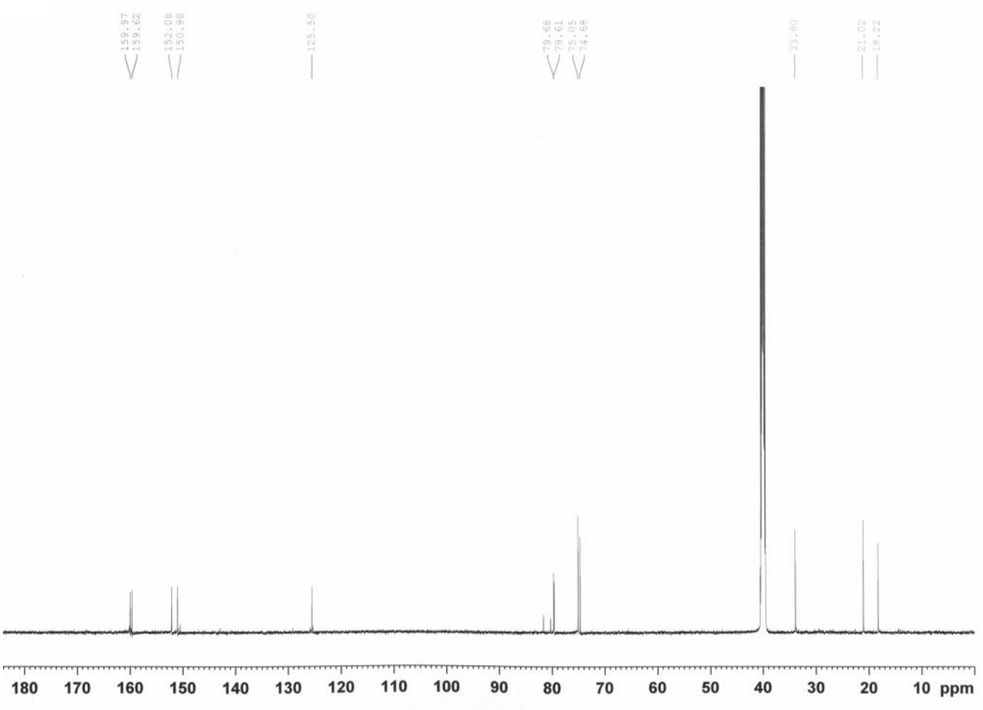

Compound **15a** HR ESIMS

Mass Spectrum List Report

Analysis Info

Analysis Name	D:\Data\AK5_1A.d	Acquisition Date	12/29/2016 9:22:24 AM
Method	low_mass.m	Operator	KM
Sample Name	TM Low concentration	Instrument	impact II 1825265.10082
Comment			

Acquisition Parameter

Source Type	ESI	Ion Polarity	Positive	Set Nebulizer	0.3 Bar
Focus	Active	Set Capillary	4000 V	Set Dry Heater	240 °C
Scan Begin	100 m/z	Set End Plate Offset	-500 V	Set Dry Gas	4.0 l/min
Scan End	1000 m/z	Set Charging Voltage	2000 V	Set Divert Valve	Source
		Set Corona	0 nA	Set APCI Heater	0 °C

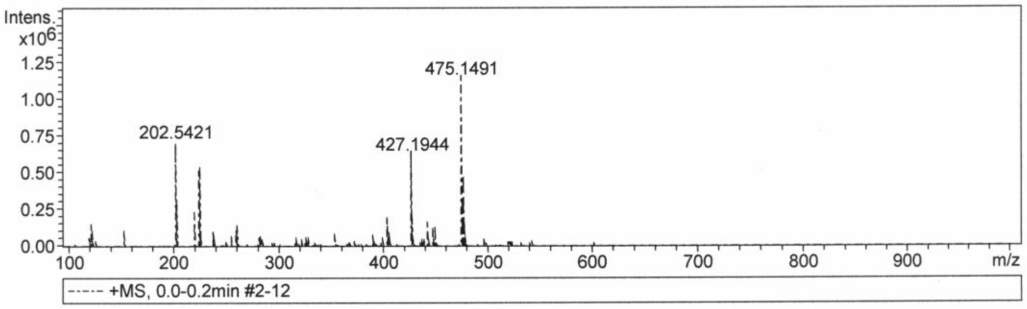

#	m/z	Res.	S/N	I	I %	FWHM
1	202.5421	27883	8840.9	703557	62.0	0.0073
2	220.0906	26954	2808.0	237875	21.0	0.0082
3	225.5160	27358	5909.8	515143	45.4	0.0082
4	427.1944	32656	4464.4	622209	54.8	0.0131
5	475.1491	35824	7917.9	1134412	100.0	0.0133
6	477.1465	30312	3349.8	480997	42.4	0.0157

Compound **15a** ^{1}H NMR (DMSOd_6)

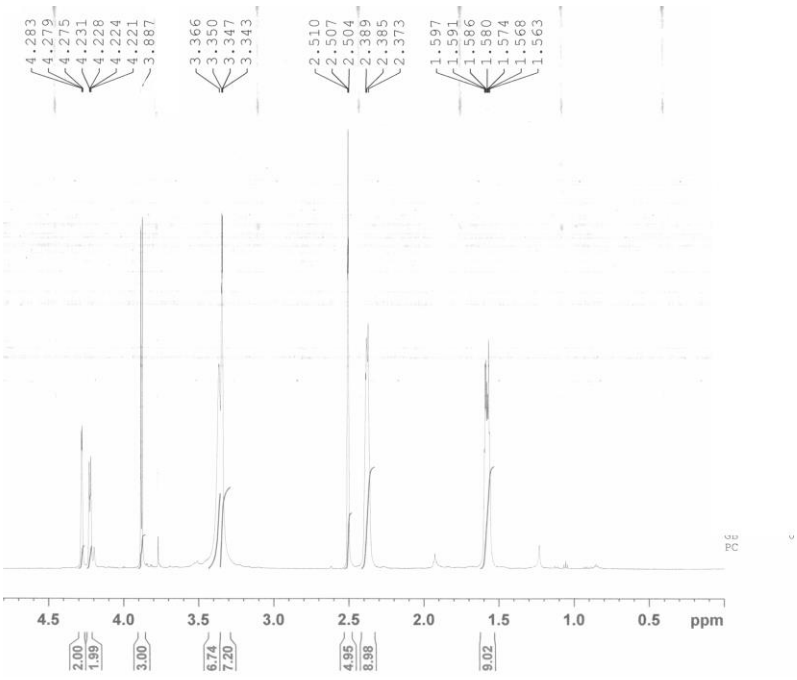

Compound **15a** ^{13}C NMR (DMSOd_6)

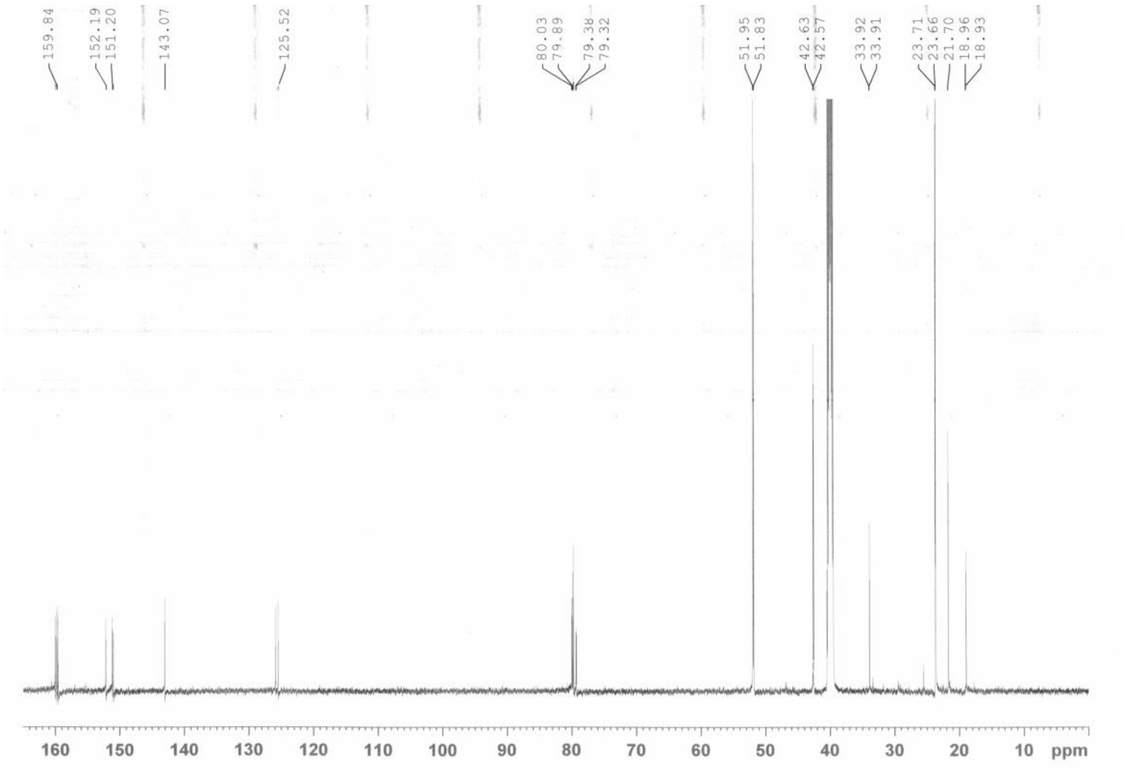

Compound **15b** HR ESIMS

Mass Spectrum List Report

Analysis Info

Analysis Name	D:\Data\PM-2016-09-20\AK6_1.d	Acquisition Date	12/16/2016 11:27:35 AM
Method	low_mass.m	Operator	KM
Sample Name	TM Low concentration	Instrument	impact II 1825265.10082
Comment			

Acquisition Parameter

Source Type	ESI	Ion Polarity	Positive	Set Nebulizer	0.3 Bar
Focus	Active	Set Capillary	4000 V	Set Dry Heater	240 °C
Scan Begin	100 m/z	Set End Plate Offset	-500 V	Set Dry Gas	4.0 l/min
Scan End	1000 m/z	Set Charging Voltage	2000 V	Set Divert Valve	Source
		Set Corona	0 nA	Set APCI Heater	0 °C

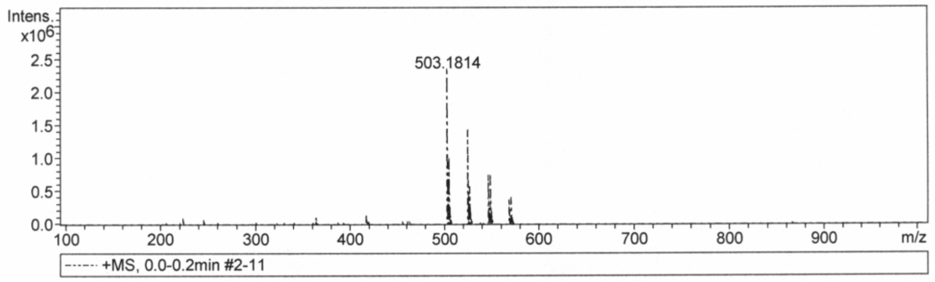

#	m/z	Res.	S/N	I	I %	FWHM
1	503.1814	42471	25597.7	2306333	100.0	0.0118
2	505.1788	35265	10410.4	948837	41.1	0.0143
3	525.1633	39587	15126.8	1440531	62.5	0.0133
4	549.1289	34825	7683.4	759218	32.9	0.0158
5	571.1110	31790	4322.3	424544	18.4	0.0180

Compound **15b** ^{1}H NMR (DMSOd_6)

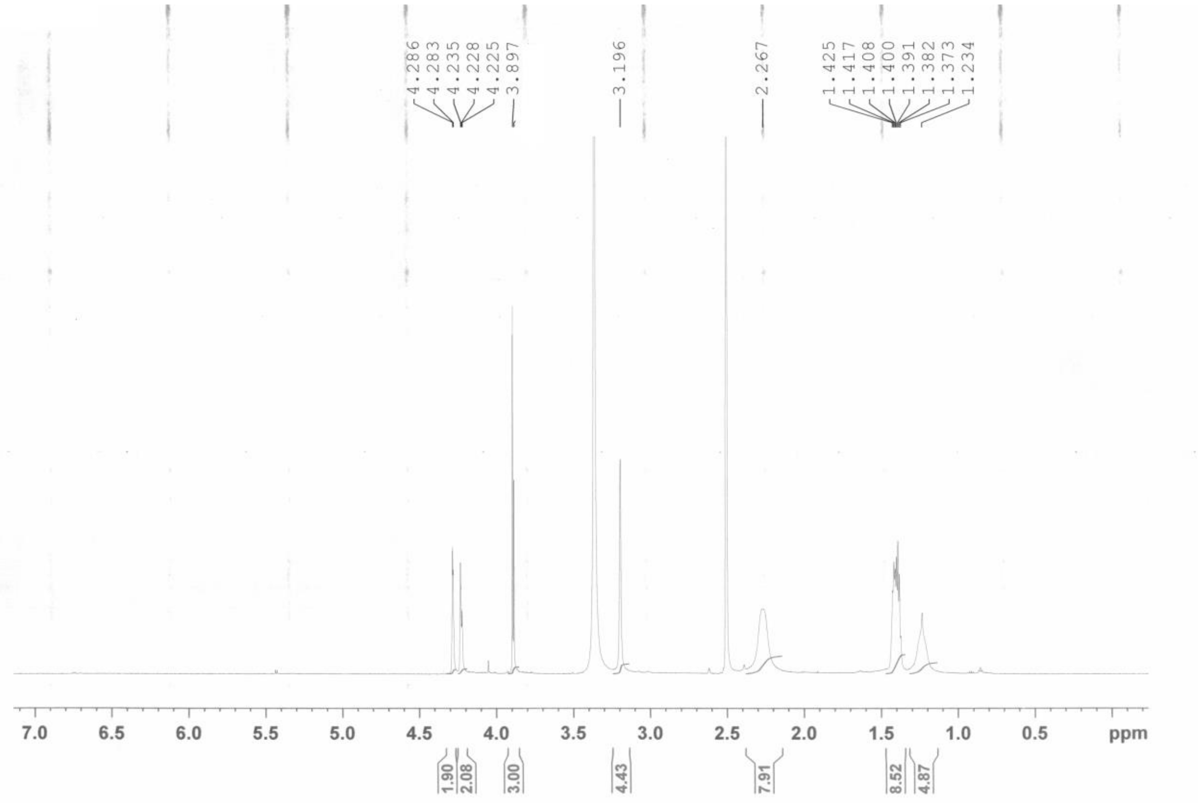

Compound **15b** ^{13}C NMR (DMSOd_6)

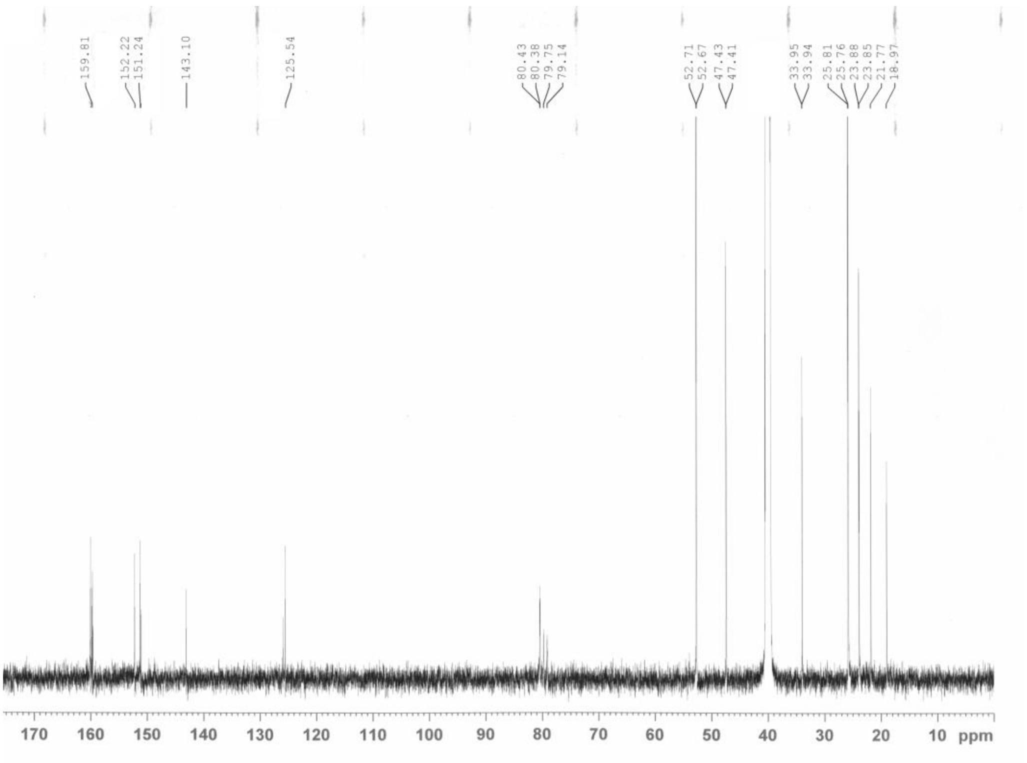

Compound **15c** HR ESIMS

Mass Spectrum List Report

Analysis Info

Analysis Name	D:\Data\PM-2016-09-20\AK8a.d	Acquisition Date	12/13/2016 12:18:28 PM
Method	low_mass.m	Operator	KM
Sample Name	TM Low concentration	Instrument	impact II 1825265.10082
Comment			

Acquisition Parameter

Source Type	ESI	Ion Polarity	Positive	Set Nebulizer	0.3 Bar
Focus	Active	Set Capillary	4000 V	Set Dry Heater	240 °C
Scan Begin	100 m/z	Set End Plate Offset	-500 V	Set Dry Gas	4.0 l/min
Scan End	2000 m/z	Set Charging Voltage	2000 V	Set Divert Valve	Source
		Set Corona	0 nA	Set APCI Heater	0 °C

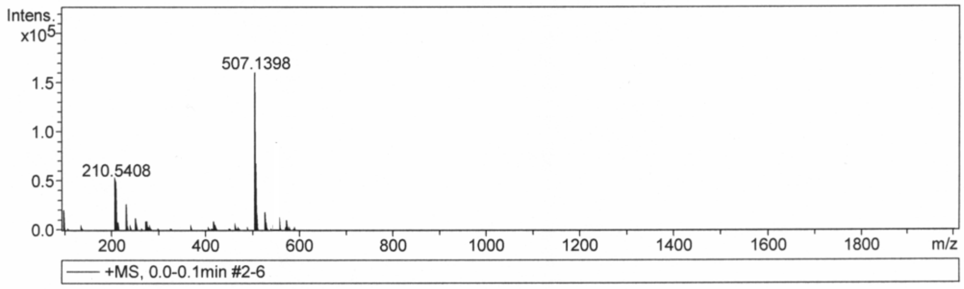

#	m/z	Res.	S/N	I	I %	FWHM
1	210.5408	24761	1553.0	51187	32.1	0.0085
2	233.5146	25028	792.1	27278	17.1	0.0093
3	507.1398	24321	3614.8	159641	100.0	0.0209
4	529.1222	23627	443.3	19399	12.2	0.0224

Compound **15c** ^{1}H NMR (DMSOd_6)

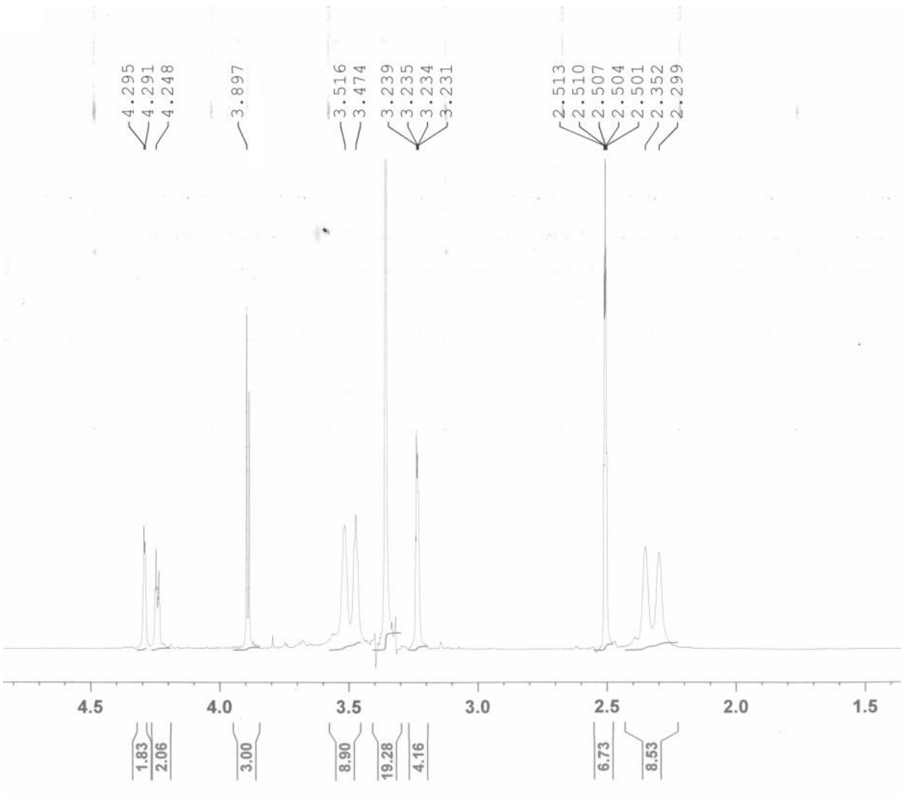

Compound **15c** ^{13}C NMR (DMSOd_6)

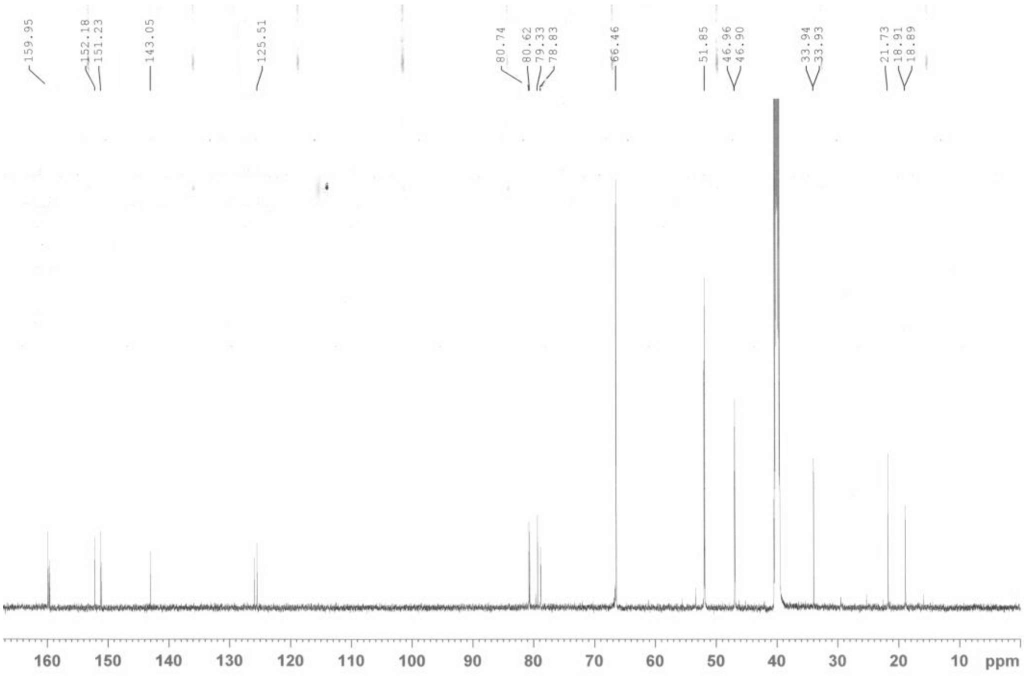

Compound **15d** HR ESIMS

Mass Spectrum List Report

Analysis Info

Analysis Name	D:\Data\PM-2016-09-20\AK7.d	Acquisition Date	12/13/2016 12:57:00 PM
Method	low_mass.m	Operator	KM
Sample Name	TM Low concentration	Instrument	impact II 1825265.10082
Comment			

Acquisition Parameter

Source Type	ESI	Ion Polarity	Positive	Set Nebulizer	0.3 Bar
Focus	Active	Set Capillary	4000 V	Set Dry Heater	240 °C
Scan Begin	100 m/z	Set End Plate Offset	-500 V	Set Dry Gas	4.0 l/min
Scan End	2000 m/z	Set Charging Voltage	2000 V	Set Divert Valve	Source
		Set Corona	0 nA	Set APCI Heater	0 °C

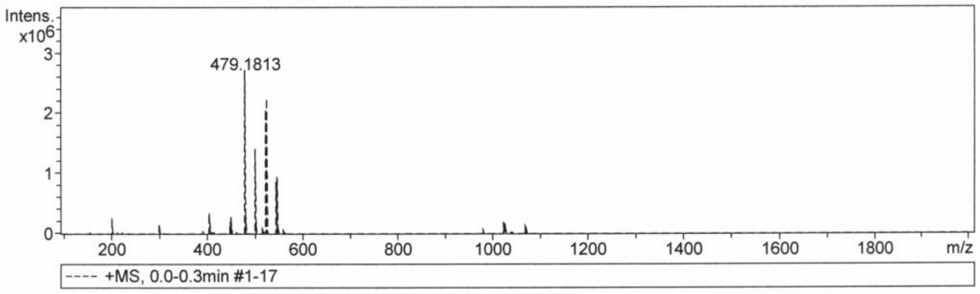

#	m/z	Res.	S/N	I	I %	FWHM
1	301.1414	29843	2534.5	175922	6.6	0.0101
2	406.0922	31936	3750.6	363021	13.7	0.0127
3	450.0416	31380	1933.5	216797	8.2	0.0143
4	479.1813	43478	19032.2	2648675	100.0	0.0110
5	501.1631	41197	8788.8	1343628	50.7	0.0122
6	525.1286	43978	14188.5	2241008	84.6	0.0119
7	547.1104	39229	6009.6	964108	36.4	0.0139

Compound **15d** ^{1}H NMR (DMSO*d*$_6$)

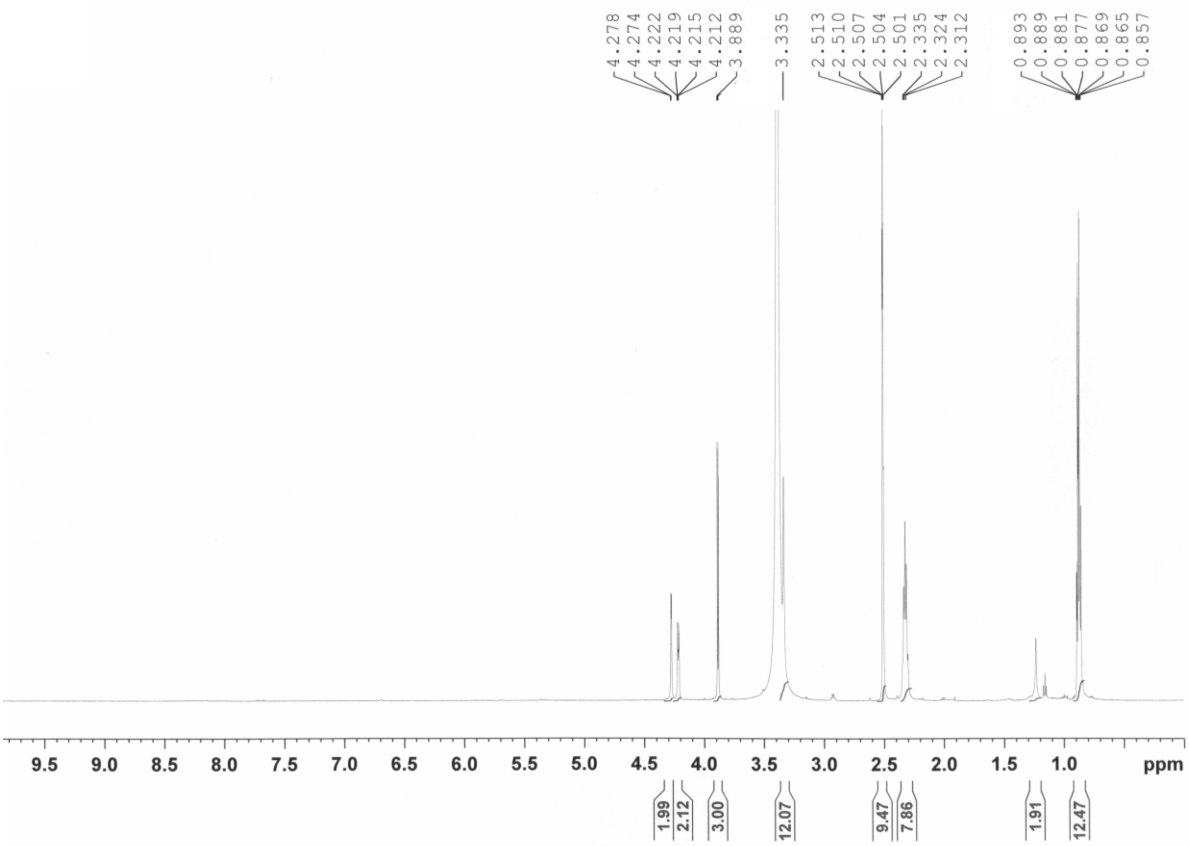

Compound **15d** ^{13}C NMR (DMSOd_6)

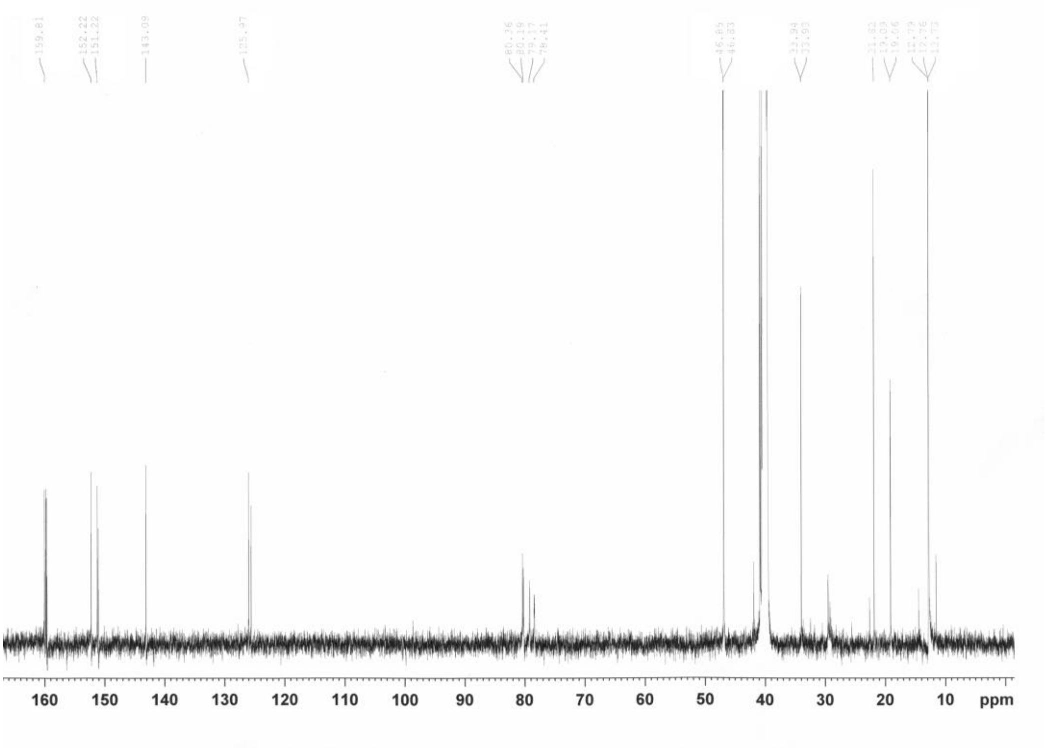

Supplement: Supplementary file 1 — Supplementary Material [file 44_2018_2155_MOESM1_ESM.pdf]
